# Insights Into Amentoflavone: A Natural Multifunctional Biflavonoid

**DOI:** 10.3389/fphar.2021.768708

**Published:** 2021-12-22

**Authors:** Xifeng Xiong, Nan Tang, Xudong Lai, Jinli Zhang, Weilun Wen, Xiaojian Li, Aiguo Li, Yanhua Wu, Zhihe Liu

**Affiliations:** ^1^ Guangzhou Institute of Traumatic Surgery, Guangzhou Red Cross Hospital, Jinan University, Guangzhou, China; ^2^ Department of Traditional Chinese Medicine, Guangzhou Red Cross Hospital, Jinan University, Guangzhou, China; ^3^ Department of Infectious Disease, Guangzhou Red Cross Hospital, Jinan University, Guangzhou, China; ^4^ Department of Burn and Plastic Surgery, Guangzhou Red Cross Hospital, Jinan University, Guangzhou, China

**Keywords:** amentoflavone, anti-cancer, anti-SARS-CoV-2, biological activity, drug delivery, molecular target

## Abstract

Amentoflavone is an active phenolic compound isolated from *Selaginella tamariscina* over 40 years. Amentoflavone has been extensively recorded as a molecule which displays multifunctional biological activities. Especially, amentoflavone involves in anti-cancer activity by mediating various signaling pathways such as extracellular signal-regulated kinase (ERK), nuclear factor kappa-B (NF-κB) and phosphoinositide 3-kinase/protein kinase B (PI3K/Akt), and emerges anti-SARS-CoV-2 effect via binding towards the main protease (Mpro/3CLpro), spike protein receptor binding domain (RBD) and RNA-dependent RNA polymerase (RdRp) of SARS-CoV-2. Therefore, amentoflavone is considered to be a promising therapeutic agent for clinical research. Considering the multifunction of amentoflavone, the current review comprehensively discuss the chemistry, the progress in its diverse biological activities, including anti-inflammatory, anti-oxidation, anti-microorganism, metabolism regulation, neuroprotection, radioprotection, musculoskeletal protection and antidepressant, specially the fascinating role against various types of cancers. In addition, the bioavailability and drug delivery of amentoflavone, the molecular mechanisms underlying the activities of amentoflavone, the molecular docking simulation of amentoflavone through *in silico* approach and anti-SARS-CoV-2 effect of amentoflavone are discussed.

## 1 Introduction

**FIGURE 1:**
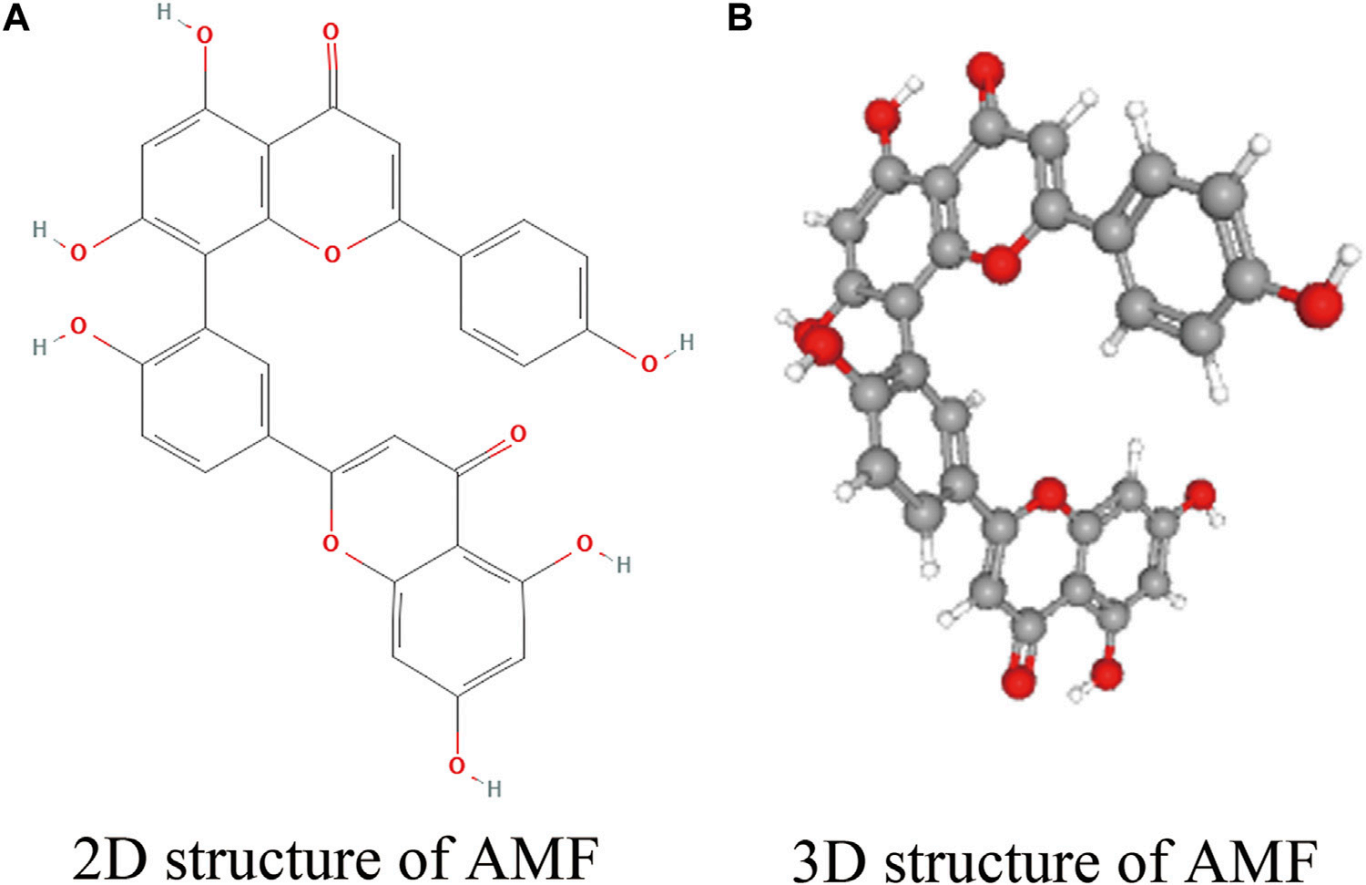
Structure of AMF. **(A)** 2D structure of AMF; **(B)** 3D structure of AMF.

Amentoflavone (AMF, Figure 1), a natural biflavonoid compound, is widely used in traditional Chinese medicine. AMF is initially isolated from the leaves of *Selaginella tamariscina, Selaginella rupestris* and *Ginkgo biloba* by Okigawa et al. (1971), Chakravarthy et al. (1981) and Lobstein-Guth et al. (1988). After that, AMF is also successively extracted from more than 120 plants (Yu et al., 2017) such as *Celaenodendron mexicanum*, *Cupressus funebris*, *Garcinia multiflora, Biophytum sensitivum, Rhus succedanea, Hypericum perforatum*, *Cupressocyparis leylandii* (Lin et al., 1997; Krauze-Baranowska et al., 1999; Camacho et al., 2000; Jurgenliemk and Nahrstedt, 2002). AMF has been shown to exhibit multiple biological activities including anti-inflammatory (Tordera et al., 1994; Kim et al., 1998; Oh et al., 2013; An et al., 2016; Cai et al., 2019), antibacterial (Hwang et al., 2013), antifungal (Jung et al., 2006; Jung et al., 2007; Hwang et al., 2012), antivirus (Lin et al., 1997; Wilsky et al., 2012; Coulerie et al., 2013), anti-oxidative (Bonacorsi et al., 2012; Li et al., 2020), anti-angiogenesis (Guruvayoorappan and Kuttan, 2008c; Tarallo et al., 2011; Zhang et al., 2014), neuroprotection (Cao et al., 2017; Chen et al., 2018; Rong et al., 2019; Zhao et al., 2019; Cao et al., 2021), osteogenesis (Zha et al., 2016), anti-arthritis (Bais et al., 2017; Vasconcelos et al., 2019), radioprotection (Park et al., 2011; Xu et al., 2014; Qu et al., 2019), antidiabetic (Qin et al., 2018; Su et al., 2019) and antidepressant (Ishola et al., 2012). It is reported that AMF exerts anti-cancer activity through a variety of mechanisms (Guruvayoorappan and Kuttan, 2007; Lee et al., 2011; Pei et al., 2012; Zheng et al., 2016; Liu et al., 2017a; Pan et al., 2017; Chiang et al., 2019; Hsu et al., 2019; Park and Kim, 2019; Chen et al., 2020b). In this review, the biological activities of AMF will be discussed comprehensively.

## 2 Chemistry of Amentoflavone

AMF, also to be known as 3′, 8″-biapigenin, belongs to the class of biflavonoids and polyflavonoids, one of organic compounds which abundantly exist in *Selaginella tamariscina* (Selaginellaceae family) with C_30_H_18_O_10_ molecular formula and a molecular weight of 538.46 g/mol. The international union of pure and applied chemistry (IUPAC) name of AMF is 8-(5-(5,7-dihydroxy-4-oxo-4H-chromen-2-yl)-2-hydroxyphenyl)-5,7-dihydroxy-2-(4-hydroxyphenyl)-4H-chromen-4-one. A registry number of the Chemical Abstracts Service (CAS) is 1617-53-4. AMF possesses a dimer of two apigenins with six hydroxyl groups on C5, C7, C4’, C5″, C7″, and C4''' positions (Yu et al., 2017). Thus, AMF is considered to be a flavonoid lipid molecule and is a very hydrophobic molecule, practically insoluble in water (0.0072 g/L at 25°C) and relatively neutral, but easily soluble in alcohol and DMSO (https://hmdb.ca/metabolites/HMDB0030832). The melting point of AMF is 300°C. The 2D and 3D structures of AMF are shown in Figure 1 (https://pubchem.ncbi.nlm.nih.gov/compound/5281600).

## 3 The Multifunctional Biological Activities of Amentoflavone

As a natural biflavonoid compound, AMF is reported to play various pharmacological effects such as anti-inflammatory (Tordera et al., 1994; Kim et al., 1998; Woo et al., 2005; Huang et al., 2012; Jeong et al., 2012; Ishola et al., 2013; Oh et al., 2013; Sakthivel and Guruvayoorappan, 2013; An et al., 2016; Trang et al., 2016; Zong and Zhang, 2017; Cai et al., 2019; Kuo et al., 2019; Alkadi et al., 2021), anti-microorganism (Lin et al., 1997; Ma et al., 2001; Jung et al., 2006; Jung et al., 2007; Ryu et al., 2010; Hwang et al., 2012; Wilsky et al., 2012; Coulerie et al., 2013; Hwang et al., 2013; Yin et al., 2014; Zhao et al., 2017b; Shen et al., 2018; Bajpai et al., 2019; Liu et al., 2020a), anti-oxidant (Bonacorsi et al., 2012; Li et al., 2020), anti-angiogenesis (Kang et al., 2004; Dell'Agli et al., 2006; Guruvayoorappan and Kuttan, 2008c; Tarallo et al., 2011; Zhang et al., 2014), neuroprotective (Kang et al., 2005; Shin et al., 2006; Zhang et al., 2015; Cao et al., 2017; Chen et al., 2018; Rong et al., 2019; Zhao et al., 2019; Liu et al., 2020b; Choi et al., 2020; Sun et al., 2020; Cao et al., 2021), musculoskeletal protection (Lee et al., 2006; Zha et al., 2016; Bais et al., 2017; Zhang et al., 2018; Vasconcelos et al., 2019), radioprotection (Lee et al., 2008; Park et al., 2011; Lee et al., 2012; Xu et al., 2014; Qu et al., 2019), metabolism regulation (Na et al., 2007; Chen et al., 2016; Yao et al., 2016; Qin et al., 2018; Su et al., 2019; Zhang et al., 2019), anxiolytic/antidepressant (Ishola et al., 2012) and anti-cancer (Banerjee et al., 2002b; Guruvayoorappan and Kuttan, 2007; Lee et al., 2009; Lee et al., 2011; Zheng et al., 2016; Liu et al., 2017a; Pan et al., 2017; Yen et al., 2018; Chiang et al., 2019; Chen et al., 2020b), etc. In addition to the anti-oxidant effect, it has also been reported that AMF can promote oxidation (Wahyudi et al., 2018). The multifunctional biological activities of AMF are detailed in Table 1.

**TABLE 1:** The mutiple biological activities of AMF.

Category	Models	Doses	Biological activities	References
Anti-inflammation	Rats’ neutrophils	4.5 ± 0.1 × 10^−5^ M, 6.2 ± 0.4×10^−4^ M	*β*-glucuronidase↓, Lysozyme release↓	Tordera et al. (1994)
	Rat carrageenan paw edema model	42 mg/kg	Group II phospholipase A2↓, Cyclooxygenase↓	Kim et al. (1998)
	LPS-induced RAW264.7 cells	0-200 μM	NO↓, PGE2↓, c-FOS↓, AP-1↓, ERK↓, iNOS↓, TNF-α↓, COX-2↓, IL-1β↓	Oh et al. (2013)
	IMQ psoriasis-like mice; HaCaT cells	25 mg/kg, 50 mg/kg; 10–20 μg/ml	TNF-α↓, IL-17↓, IL-22↓, IL-23↓, Cyclin D1↓, Cyclin E↓, NF-κB p65↓	An et al. (2016)
	SD rats	15 mg/kg, 30 mg/kg	C3↓, BCR/NF-κB signaling pathway↓, HMGB1↓	Cai et al. (2019)
	LPS-induced RAW264.7 cells	3, 10, 30 and 60 μM	iNOS↓, NF-κB p65↓, I-κBα degradation↓	Woo et al. (2005)
	LPS-induced RAW264.7 cells	1, 10 and 100 μM	NO↓	Jeong et al. (2012)
	LPS-induced RAW264.7 cells	0.03 μM	PGE2↓, NO↓, SOCS3↑, TNF-α↓, IL-6↓, IL-1β↓	Huang et al. (2012)
	Rats with ulcerative colitis	10 mg/kg	LDH↓, MPO↓, LPO↓, GSH↑, SOD↑, NO↓, TNF-α↓, COX-2↓, IL-1β↓, iNOS↓, IL-6↓, NF-κB p65/p50↓	Sakthivel and Guruvayoorappan, (2013)
	LPS-induced C6 cells, LPS-induced THP-1 cells, SD rats	0.1–3 μg/ml, 6.25–50 mg/kg	Nirtite release↓, ROS↓, MDA↓, TNF-α↓, GSH↑, Reduce number of writhes, Increase pain threshold, Decrease oedema formation	Ishola et al. (2013)
	LPS-stimulated BV2 cells, LPS-stimulated RAW264.7 cells	IC_50_: 12.4 ± 2.1 μM (BV2 cells); 19.8 ± 3.3 μM (RAW264.7 cells)	NO↓	Trang et al. (2016)
	CLP-induced septic rats	50 mg/kg	TNF-α↓, IL-1β↓, GSH↑, SOD↑, NF-κB p65↓, TBARS↓, Nrf2↑,GCLc↑	Zong and Zhang, (2017)
	LPS-induced RAW264.7 cells	5, 10 μg/ml	NO↓	Kuo et al. (2019)
	THP-1 cells	0-30 μg/ml	PGE2↓, IL-6↓, TNF-α↓	Alkadi et al. (2021)
Anti-microorganism	CVB3-infected Raji cells	25–200 μM	Reduce coxsackievirus B3 replication, Inhibit FAS activity	Wilsky et al. (2012)
	*S. aureus, E. faecium, P. aeruginosa, S. mutans, E. coli, E. coli-157*	4, 8, 16, 32 μg/ml	NADH depletion	Hwang et al. (2013)
	Mouse Gas Gangrene model, CPA-treated Caco-2 cells, PFO-treated Caco-2 cells	50 mg/kg, 0–16 μg/ml, 0–16 μg/ml	Block the hemolysis and cytotoxicity induced by CPA and PFO, Survival rates↑, Survival time↑, LDH release↓, CPA-mediated virulence↓, PFO-mediated virulence↓	Liu et al. (2020a)
	SARS-CoV 3CL^pro^ inhibition assay	IC50: 8.3 ± 1.2 μM	Inhibit SARS-CoV 3CL^pro^ activity	Ryu et al. (2010)
	*C.albicans, S.cerevisiae, T.beigelii*	MIC: 5 μg/ml, 5–10 μg/ml	Induced the accumulation of intracellular trehalose, Disrupt the dimorphic transition	Jung et al. (2006)
	*C.albicans*	5 μg/ml	Induce S-phase arrest	Jung et al. (2007)
	*C.albicans*	5 μM	Mitochondrial dysfunction, Induce apoptotic cell death	Hwang et al. (2012)
	Respiratory syncytial virus (RSV)	IC_50_: 5.5 μg/ml		Ma et al. (2001)
	HIV-1 RT	IC_50_: 119 μg/ml		Lin et al. (1997)
	DENV-NS5 RdRp	IC50: 1.3 ± 0.1 μM	Inhibition of DENV-NS5 RdRp	Coulerie et al. (2013)
	CBV3-infected HEp-2 cells	6.25-50 μg/ml	Virucidal activity↑	Yin et al. (2014)
	Pneumolysin (PLY)-mediated A549 cells, *S.pneumoniae strain* D39- infected mice	0–64 μg/ml, 50 mg/kg	Weaken hemolytic activity of PLY, Weaken PLY-mediated A549 cell injury, Reduce the virulence of PLY	Zhao et al. (2017b)
	*S.suis*-infected mice, *S.suis serotype 2 (SS2)*-infected J774 cells	100 mg/kg, 0–32 μg/ml	TNF-α↓, IL-6↓, IL-1β↓, Lower mortality and bacterial burden, p38↓, JNK1/2↓, NF-κB p65↓, SLY pore-forming activity↓	Shen et al. (2018)
	*S.aureus, E.coli*	MIC: 62.5 μg/ml, 125 μg/ml	K+ release↑, ATP release↑	Bajpai et al. (2019)
	HCV-infected Huh-7 cells	1–100 μM	Inhibit HCV RNA replication, Circumvent daclatasvir-induced RAVs (Resistance-Associated Variants)	Lee et al. (2018c)
	HSV-1 infected Vero cells, HSV-1 infected SK-N-SH cells	2.5-50 μM	*UL54 gene*↓, *UL52 gene*↓, *UL27 gene*↓, ICP0↓, gD↓, VP5↓, Inhibit ACV-resistant strains, Reduce the nuclear transport	Li et al. (2019a)
	*M.aeruginosa*	32–512 μg/ml	Ameliorate cell membranes, peptidoglycan layers and cytoplasm, HCB control agent	Lee et al. (2020)
	*L.amazonensis-*infected peritoneal macrophages, *L.amazonensis-*infected mice	0–11.4 μM, 0.05 mg/kg	NO↓, iNOS↓, HO-1↓, Nrf2↓, Ferritin↑, ROS↑	Rizk et al. (2021)
Pro-oxidation	HaCaT cells	0-100 μM	Nrf2↑, ARE↑, NQO-1↑, ROS↑, p-p38↑, p-AKT↑, p-ERK1/2↑	Wahyudi et al. (2018)
Anti-oxidation	H_2_O_2_-induced HL-O2 cells	62.5, 125, 250 μM	ROS↓, Trx1↑, TrxR1↑, ASK1↓, p-p38↓, SOD↑, ALT↓, AST↓, LDH↓, MDA↓, MMP↑, Cyt-C↓, Caspase 9↓, Caspase 3↓	Li et al. (2020)
	*H. pylori*-induced PMNs	0–100 μg/ml, IC50:92.9 μg/ml	ROS↓	Bonacorsi et al. (2012)
Neuroprotection	H_2_O_2_-induced SH-SY5Y cells, SNP-induced SH-SY5Y cells, Aβ25-35-induced PC12 cells, Etoposide-induced SH-SY5Y cells	0.4–10 μM	Oxidative stress↓, Aβ↓, DNA-damage↓	Kang et al. (2005)
	Neonatal H-I rat brain injury model, LPS-induced BV-2 cells	10 mg/kg *in vivo*, 30 mg/kg *in vivo*, 0-50 μM *in vitro*	Caspase3↓, PARP↓, α-Spectrin↓, Procasp 3↓, p35↑, iNOS↓, COX-2↓, IL-1β↓, TNF-α↓, OX-42↓	Shin et al. (2006)
	MPTP-induced mice, MPP^+^-treated SH-SY5Y cells	30 mg/kg *in vivo*, 0-150 μM *in vitro*	Cleaved-caspase3↓, p21↓, Bcl-2/Bax↑, p-PI3K↑, p-AKT↑, p-ERK1/2↑, IL-1β↓, iNOS↓, tyrosine hydroxylase↑, GFAP↓, Iba1↓	Cao et al. (2017)
	Aβ_1-42_-injected AD Rats, Aβ_1-42_-treated PC12 cells	40, 80 mg/kg *in vivo*, 10, 20 μM *in vitro*	Nrf2↑, p-AMPK↑, p-GSK3β↑, HO-1↑, NQO-1↑, Cleaved-caspase3↓	Chen et al. (2018)
	PTZ-induced kindling mice, LPS-induced BV2 microglial cells	10 μM *in vitro*, 50 mg/kg *in vivo*	NLRP3↓, ASC↓, Caspase 1↓, IL-18↓, TNF-α↓, IL-1β↓	Rong et al. (2019)
	SH-SY5Y cells, Aβ_1-42_-injected Rats	40 mg/kg and 80 mg/kg *in vivo*, 0–20 μM *in vitro*	NLRP3↓, ASC↓, Cleaved-Caspase 1↓, GSDMD↑, GSDMD-N↓, IL-18↓, IL-1β↓, p-AMPK↓, p-GSK3β↓	Zhao et al. (2019)
	BV-2 cells	0-100 μM, IC50:8.03 μM	Cell cycle arrest at G2/M, CDK2↑, p27↑, p-p53↑, CDK1/CDC2↓, CyclinB1↓, Bax↑, c-caspase 3↑, c-caspase 9↑, BCL-XL↓, Beclin1↑, LC3↑, p-PI3K↓, p-ERK1↓	Liu et al. (2020b)
	Recombinant human Aβ_1-42_ peptide	IC50: 0.26 ± 0.03 μM, EC50: 0.59 ± 0.19 μM	Inhibit Aβ_1-42_ fibrillization, Disassemble preformed Aβ_1-42_ fibrils	Choi et al. (2020)
	Transgenic 5xFAD mice, Aβ_42_ fibrils-treated neuro2A cells	25 μM	Inhibit Aβ_42_ fibrillization, Inhibit Aβ_42_ aggregation, Disaggregate Aβ_42_ fibrils, Chelate Cu^2+^, Diminish the Cu^2+^-ascorbate redox cycling and ROS formation	Sun et al. (2020)
	Pilocarpine-induced epilepsy mice	25 mg/kg	NF-κB activation↓, NO↓, PEG2↓, IL-1β↓, IL-6↓, reduce seizures, decrease damage and apoptosis with hippocampal neurons	Zhang et al. (2015)
	Aβ_25-35_-induced mice, PC-12 cells, APPswe-N2a cells	20 mg/kg, 5, 10 μmol/L	Aβ_42_/Aβ_40_↓, p-Tau↓, IL-6↓, IL-17↓, TNF↓, ROS↓, MDA↓, GSH-Px↑, T-SOD↑, Bax↓, Bcl2↑, caspase9↓, caspase3↓, LC3B↑, p62↓, Beclin-1, p-mTOR↓	Cao et al. (2021)
Musculoskeletal protection	hMSCs, Zebrafish larvae	0.1–10 μM *in vitro*, 0.1-5 μM *in vivo*	Runx2↑, Osx↑, p-p38↑, p-JNK↑	Zha et al. (2016)
	Wear debris-induced osteolysis mice, BMMs	20 mg/kg and 40 mg/kg *in vivo*, 0.1–10 μM *in vitro*	Inhibit F-actin rings formation, Suppress osteoclastic bone absorption, Inhibit osteolysis, p-ERK↓, p-JNK↓, p-p38↓, p-IκBα↓, c-FOS↓, NFATc1↓	Zhang et al. (2018)
	Mouse osteoblasts	1, 10, 20 μM	ALP activity↑, Collagen synthesis↑, mineralization↑	Lee et al. (2006)
	CFA-induced arthritic rats	20 mg/kg and 40 mg/kg	SGOT↓, SGPT↓, ALP↓, TNF-α↓, ESR, HB↑	Bais et al. (2017)
	MIA-induced OA rats	50, 150, 450 mg/kg	COX-1↓, COX-2↓	Vasconcelos et al. (2019)
Radioprotection	UV irradiated- human skin fibroblasts	1.25–5 μM, IC50:1.8 μM	MMP-1↓	Lee et al. (2008)
	UV irradiated- human skin fibroblasts	1.25-5 μM	MMP-1, p-ERK, p-c-Jun, c-Fos	Lee et al. (2012)
	UVB-irradiated fibroblasts	1.25, 2.5, 5 μM	LaminA↓, p-H2AX↓, Progerin↓, actin↑	Park et al. (2011)
	γ-irradiation- induced mice	0.24, 1.2, 6 mg/kg	TNFAIP2↑, CFU-GM↑, Micronucleus frequency↓, SOD↑, GSH↑	Qu et al. (2019)
	γ-ray-irradiated V79 cells	1-12 μg/ml	ROS↓, mitochondrial mass↓, cells of G2 phase↑	Xu et al. (2014)
Metabolism regulation	32D cell overexpressing IR	IC50 7.3 ± 0.5 μM, 0.1–10 μM	PTP1B↓, Tryrosine phosphorylation of IR↑	Na et al. (2007)
	LPS-induced HUVECs	4.647, 9.294, 18.587 μM	NO↓, MDA↓, SOD↑, glutathione metabolism↑, Putrescine↑, Spermidine↑, 5-oxoproline↑, Arginine ardproline metabolism↑	Yao et al. (2016)
	3T3-L1 pre-adipocytes, High-fat diet-rats	10, 50 mg/kg, 1, 5, 10 μg/ml	FBG↓, FI↓, BW↓, PATW↓, TG↓, C/EBPB↓, ROS↑, PPARγ↓, MCE↓, Inhibition of adipocyte differentiation	Chen et al. (2016)
	High fructose and fat diet-induced MS rats	100 mg/kg	PE↓, Ach↑, NO↑, AT-1A↓, AT-2↑, TBARS↓, GSH↑, SOD↑, Catalase↑, NADPH oxidase activity↓	Qin et al. (2018)
	Diabetic mice	20, 40 mg/kg	Glucose↓, TC↓, TG↓, LDL-C↓, glucagon↓, HDL-C↑, insulin↑, GCK↑, PK↑, PFK-1↑, GSK3↓, SOD↑, PEPCK↓, MDA↑, G-6-pase↓, p-Akt↑, GLUT4↑	Su et al. (2019)
	KKAy mice	0.2 g/kg	TNF-α↓, hs-CRP↓, TG↓, FFA↓, LDL-C↓, HDL-C↓, PPARγ↑, Glu-2↑, Foxo1↓, PI3K/Akt signaling↑	Zhang et al. (2019)
Anxiolytic/antidepressant	Swiss albino mice	6.25-50 mg/kg	Bind to GABA receptor, Interact with 5-HT2 receptor, Interact with α1-andα2-adrenoceptors, Increase number of head-dips	Ishola et al. (2012)

### 3.1 Anti-inflammatory Activity

Inflammation is a natural defense mechanism that protects the human body from a variety of infections (Ellis, 2001). However, the development of inflammatory diseases such as bronchitis, gastritis, enteritis, rheumatoid arthritis and psoriasis is often caused by chronic inflammation (Kaplanski et al., 2003). Kinds of diseases have been attempted to be treated by flavonoids as an anti-inflammation agent. Tordera et al. (1994) demonstrate that the anti-inflammatory activity of AMF can affect neutrophil function through inhibiting *β*-glucuronidase and lysozyme basal release in rat neutrophils. AMF also shows a potential anti-inflammatory activity through the inhibition on activities of group II phospholipase A2 and cyclooxygenase in the rat carrageenan paw edema model (Kim et al., 1998). AMF treatment decreases the inflammatory activation of mouse microglial cells after hypoxic-ischeamic (H-I) injury (Shin et al., 2006). AMF could ameliorate IMQ-induced psoriasis-like skin lesion in mice by decreasing NF-κB-mediated inflammation and keratinocyte proliferation (An et al., 2016). In addition, AMF shows anti-inflammatory activity *via* suppressing LPS-induced NO and PGE2, the inhibition of iNOS and COX-2 expression, and the inhibition of NF-κB signaling pathway in macrophages (Woo et al., 2005; Hammer et al., 2007; Huang et al., 2012; Jeong et al., 2012; Tsai et al., 2012; Oh et al., 2013; Trang et al., 2016; Li et al., 2019b; Kuo et al., 2019). AMF significantly attenuates LPS-induced nitrite release, ROS, MDA formation and TNF-a generation and also upregulates the level of GSH on C6 and THP-1 cells (Ishola et al., 2013; Alkadi et al., 2021). AMF can ameliorate the inflammatory response to cold exposure-stimulated lung tissue by inhibition of C3, HMGB1 and BCR/NF-κB signaling pathway (Cai et al., 2019).

### 3.2 Anti-Microorganism Activity

Infectious disease caused by pathogenic microorganisms affects millions of people worldwide (Hwang et al., 2013). Several studies have reported that AMF is a new strategy for treating microorganism infections, including antiviral (Ma et al., 2001), antifungal (Jung et al., 2006), anti-bacterial (Zhao et al., 2017b) and antileishmanial activity (Rizk et al., 2014; Rizk et al., 2021).

Upper respiratory infection is a common disease worldwide, which is majorly caused by respiratory syncytial virus (RSV) (Borchers et al., 2013). Ma et al. (2001) report that AMF shows potent antiviral activity against RSV, with an IC_50_ of 5.5 mg/ml. Besides that, it is reported that AMF has antiviral activity against Coxsackievirus B3 (Wilsky et al., 2012; Yin et al., 2014), Dengue virus (Coulerie et al., 2013), Hepatitis C virus (HCV) (Lee et al., 2018c), Herpes Simplex Virus type 1 (HSV-1) (Li et al., 2019a), and SARS-CoV (Ryu et al., 2010). Wilsky et al. (2012) demonstrate that CVB3 infection induces an up-regulation of FAS expression, while the inhibition of FAS expression by AMF inhibits CVB3 replication in human Raji cells. Yin et al. (2014) find that AMF prevents the cytopathic effect (CPE) of CVB3 in HEp-2 cells, and significantly reduces mean viral titers in the heart and kidney which are infected with CVB3 in KM mice. Dengue virus is a prevalent human pathogenic arbovirus (WHO, 2009), the non-structural protein NS5 of which is essential for virus replication (Masse et al., 2010). Coulerie et al. (2013) demonstrate that AMF was a strong and specific noncytotoxic inhibitor of the Dengue virus NS5 RNA-dependent RNA polymerase (DENV-NS5 RdRp). Hepatitis C virus (HCV) is recognized as a major causative agent of chronic hepatitis, cirrhosis, and hepatocellular carcinoma (Kuo et al., 1989). Lee et al. (2018c) identify that AMF inhibited viral entry, replication, and translation of the HCV life cycle, and also exhibits inhibitory effects on resistant-associated variants to the NS5A inhibitor daclatasvir. Herpes Simplex Virus type 1 (HSV-1) is a DNA virus and belongs to α subfamily herpesviridae, which can cause many clinical disorders (i.e., keratitis and encephalitis) (Widener and Whitley, 2014). Li et al. (2019a) reveal that the anti-herpes viral activity of AMF toward HSV-1 and ACV-resistant strains mainly impairs HSV-1 early infection. Furthermore, AMF affects cofilin-mediated F-actin reorganization, decreases the cell membrane transport to the nucleus of HSV-1, and reduces of viral-immediate genes transcription (Li et al., 2019a). SARS-CoV, a positive-strand RNA virus, encodes a chymotrypsin-like protease (3CLpro), which plays a pivotal role in controlling replicase complex activity and processing viral polyproteins (Anand et al., 2003). Ryu et al. (2010) confirm that AMF is an effective inhibitor of SARS-CoV 3CLpro.

Also, AMF exhibits potent antifungal activity in energy-independent manner by significantly arresting cell cycles at S-phase in human pathogenic fungi *C. albicans* (Jung et al., 2006; Jung et al., 2007). As well as Jung’s results, Hwang et al. (2012) demonstrate that promoting programmed cell death is one antifungal mechanism of AMF in *C. albicans* through mitochondrial dysfunction including phosphatidylserine exposure, DNA and nuclear fragmentation, intracellular ROS accumulation, and metacaspases activities. In addition, AMF reduced mitochondrial inner-membrane potential and induced cyto-c releases (Hwang et al., 2012).

The findings of plenty researches support that AMF has considerable antibacterial activity against *S. pneumoniae*, *S. suis*, *M. aeruginosa*, *S. aureus* and *E. coli*. *S. pneumoniae* is well known as a human bacterial pathogen (Jedrzejas, 2001). As a devastating protein toxin, pneumolysin (PLY) from *streptococcus* pneumoniae punctures the cytomembrane and leads to pathological reactions such as cell disruption and inflammation (Zhao et al., 2017b). Zhao et al. (2017b) demonstrate that AMF can weaken the PLY oligomerization process by interacting with Ser254, Glu277, Arg359 sites of the toxin and confer protection against PLY-mediated injury to human alveolar epithelial cells. *Streptococcus suis* is an important zoonotic pathogen and can lead to considerable economic losses in the swine industry (Haas and Grenier, 2018). Suilysin (SLY) is a secreted extracellular pore-forming toxin which can cause necrosis, apoptosis and cell lysis in various host cells (Fittipaldi et al., 2012). AMF effectively inhibits SLY oligomerization and reduces *S. suis*-induced cytotoxicity in macrophages. Additionally, AMF reduced inflammation in *S. suis*-infected cells by regulating the p38, JNK1/2 and NF-κB pathways (Shen et al., 2018). Moreover, Lee et al. (2020) find that AMF exhibits a powerful and selective killing effect on *M. aeruginosa* without harming other non-cyanobacteria. Bajpai et al. (2019) advocate that the antibacterial effects of AMF improves the nutritional quality of minced chicken meat and apple juice through its ability to alter cell membrane permeabilities of *S. aureus* and *E. coli*. In addition, Hwang et al. (2013) reveal that the antibacterial effect of AMF and its synergistic capacity with antibiotics are mainly from the induction of hydroxyl radicals and NADH depletion.

Leishmaniases are a complex of infectious diseases caused by protozoan parasites of the genus Leishmania transmitted by the bite of sandflies (Rizk et al., 2014; Rizk et al., 2021). AMF showed a leishmanicidal action on intracellular amastigote forms, independent of NO production (Rizk et al., 2014). In infected mice, the antileishmanial activity of amentoflavone has already been reported, the mechanisms involved in the parasite death of which increased ferritin expression, ROS production, and decreased NO and iNOS expression (Rizk et al., 2021).

### 3.3 Anti-Oxidative/Pro-Oxidation Activity

Oxidative stress has been manifested to be caused by the abnormal accumulation of reactive oxygen species (ROS) and reactive nitrogen species (RNS) and promotes aging and various diseases because of the oxidative damage of liposomes, nucleic acid and proteins (Pham-Huy et al., 2008; Schieber and Chandel, 2014).

Recently, Zong and Zhang (2017) report that AMF prevents acute lung injury due to Nrf2-GCLC-via oxidative stress in septic rats. Bajpai et al. (2019) also confirm that AMF exhibits an enormous antioxidant ability by inhibiting the production of hydroxyl radicals, superoxide, ABTS and DPPH in a variety of free radical scavenging models *in vitro*. The results of Li et al. (2020) suggest that the antioxidant protection of AMF blocks ASK1/p38 MAPK pathway and alleviates hepatotoxicity in H_2_O_2_-induced HL-O2 cells by decreasing ROS generation. Bonacorsi et al. (2012) confirm that the AMF attenuates the effects of neutrophil generated ROS on gastric mucosa damage by inhibiting the oxidative burst of H. pylori-induced PMNs in gastric ulcers.

However, Wahyudi et al. (2018) reveal that AMF exhibits the prooxidative activity through the Nrf2 activation induced by ROS-mediated the activation of p38-AKT pathway in HaCaT cells. In addition, AMF plays key role in the oxidant/antioxidant balance by suppressing the production of inflammatory mediators (i.e., NO, COX-2) and pro-inflammatory cytokines (i.e., TNF-α, IL-1β and IL-6), and the activation of NF-κB signaling pathways *in vitro* or/and *in vivo* (Ishola et al., 2013; Sakthivel and Guruvayoorappan, 2013).

### 3.4 Neuroprotective Activity

The neuroprotective effect of AMF is evident in its ability to against neurodegenerative diseases, including ischemic stroke (Shin et al., 2006), epilepsy (Zhang et al., 2015), Parkinson’s disease (Cao et al., 2017) and Alzheimer’s disease (Sasaki et al., 2015; Chen et al., 2018; Sabogal-Guaqueta et al., 2018).

Hypoxic-ischemic (H-I) brain injury occurs in infants and children, which leads to permanent neurological dysfunction including learning disabilities, seizure disorders, cognitive impairment and cerebral palsy (Ashwal and Pearce, 2001). Shin et al. (2006) reveal that AMF protects the brain against H-I injury by blocking multiple molecular events which can lead to neuronal cell death. Mechanistically, AMF blocks apoptotic cell death *via* reducing the activation of caspase 3 and PARP after H-I injury.

Epilepsy is a common neurological disorder, which is characterized by recurrent and usually unprovoked epileptic seizures (Chang and Lowenstein, 2003). AMF effectively prevents the occurrence of seizures and diminishes the damage and apoptosis happening within hippocampal neurons through suppressing NF-κB signaling pathway and the production of inflammatory mediators (i.e., NO, PGE2, IL-1β and IL-6) (Zhang et al., 2015).

Parkinson’s disease (PD) is a progressive neurodegenerative disorder in the elder. PD is characterized by the degeneration of dopaminergic neurons and depletion of dopamine (DA), results in clinical symptoms of tremor, resting, bradykinesia and rigidity (de Lau and Breteler, 2006). Cao et al. (2017) disclose that AMF protects dopaminergic neurons against MPTP/MPP + -induced neurotoxicity through the activation of PI3K/Akt and ERK signaling pathways in dopaminergic neurons and the attenuation of neuroinflammation.

Alzheimer’s disease (AD) is a common progressive neurodegenerative disorder of the central nervous system, which is characterized by the deposition of amyloid *β* (Aβ) peptides as senile plaques and neurofibrillary tangles on neuronal cells (Baglietto-Vargas et al., 2016). Sasaki et al. (2015) find that AMF effectively protected PC-12 cells from Aβ42-induced cytotoxic injury by inhibiting the activation of β-secretase and reducing oxidative damage. Sabogal-Guáqueta et al. (2018) demonstrate that the treatment with AMF reduces Aβ deposition, tau pathology, microgliosis, and astrogliosis *via* the reduction of Aβ_1-40_, Aβ_1-42_ and CTFβ in the brains of aged 3xTg-AD mice. Additionally, Chen et al. (2018) reveal that AMF exerts a protective effect against Aβ_1-42_-induced deficits by modulating Nrf2 expression via AMPK signaling activation.

### 3.5 Musculoskeletal Protection

Musculoskeletal diseases (MSDs) are believed as one of the highest economic burdens to individuals and social-care systems (Woolf and Pfleger, 2003; Hoy et al., 2014). MSDs include osteoporosis (OP), rheumatoid arthritis (RA), osteoarthritis (OA), psoriatic arthritis (PsA), lower back pain (LBP) and gout (Lewis et al., 2019).

OP is known to occur due to a reduction in bone formation by osteoblasts and an increase in bone resorption by osteoclasts (Lee et al., 2006). Lee et al. (2006) report firstly in mouse osteoblasts that AMF significantly increases osteoblast differentiation by increasing alkaline phosphatase (ALP) activity and collagen synthesis, and results in mineralization. Zha et al. (2016) find that AMF significantly enhances cell proliferation, ALP activity and mineralization *via* increasing the levels of p-JNK and p-p38 in human mesenchymal stem cells (hMSCs). When the JNK and p38 MAPK pathways are inhibited by its inhibitors, the AMF-induced increases of ALP and mineralization are significantly lessened.

OA is a generally slow progression disease in which the inflammation plays a pivotal role in its pathogenesis (Wang et al., 2018). OA is characterized by pain, synovial inflammation, progressive destruction of articular cartilage, changes in the subchondral bone and peri-articular muscle (Robinson et al., 2016). Zhang et al. (2018) demonstrate the inhibition of AMF on osteoclast generation and wear debris-induced osteolysis *in vitro* and *in vivo*. AMF suppresses osteoclastogenesis, F-actin ring formation and bone absorption *in vitro*, and prevents titanium wear debris-induced osteolysis *in vivo via* suppressing the MAPKs and NF-κB pathways (Zhang et al., 2018). Also, Vasconcelos et al. (2019) suggest that AMF reduces the inflammatory process and improves OA through an interaction with cyclooxygenase-2.

RA is one of the most common inflammatory rheumatic diseases and is characterized by the development of a chronic inflammatory proliferation of the synovial linings of diarthrodial joints, which leads to aggressive cartilage destruction and progressive bony erosions (Lee and Weinblatt, 2001). Bais et al. (2017) reveal that AMF possesses potentially anti-arthritic activity *via* improvement of joint activity, decreases the paw volume and reduces the serum inflammatory TNF-a level and other RA symptoms (i.e., joint stiffness, nodules, etc) in the adjuvant induced RA rats.

### 3.6 Radioprotection

Ultraviolet (UV) radiation causes the skin to age, which is commonly related to increased sagging, wrinkling and laxity (Jenkins, 2002). This skin aging can be attributed to extrinsic (known as photo-aging) and intrinsic aging (natural-aging) (Chung et al., 2001). Alterations in the extracellular matrix (ECM) of dermis layer are observed in extrinsic aged skin by repeated exposure to UV light (Kligman, 1989; Chung et al., 2001). UV irradiation induces the synthesis of MMPs in human skin *in vivo*, and MMPs-mediated collagen destruction accounts for the connective tissue damage that occurs in aging (Rittie and Fisher, 2002). Lee et al. (2008) find that AMF could inhibit the expression of MMP-1 in human dermal fibroblasts and this might be associated with the potent NO blocking effect of AMF. Moreover, the treatment of AMF blocks the up-regulation of UVB-induced MMP-1 via the suppression of the ERK pathway and the reduction of phosphorylated c-Jun and c-Fos protein expression (Lee et al., 2012). Park et al. (2011) suggest that AMF inhibits effectively UVB-induced nuclear aberration and DNA damage through the decrease of Lamin A or phospho-H2AX protein in normal human fibroblast.

Ionizing radiation is ubiquitous in modern life and can cause mitochondrial dysfunction by inducing mitochondrial membrane damage, the reduction of the cell’s energy supply and the activation of the mitochondrial membrane potential (Xu et al., 2014). The protective effect of AMF against ionizing irradiation is investigated in irradiated v79 cells (Xu et al., 2014) and γ-irradiated mice (Qu et al., 2019). Xu *et al* reveal that the pretreatment with AMF 24 h prior to 8Gy^60^Co γ-ray irradiation treatment increases the G2 phase, inhibits apoptosis, and decreases the concentration of ROS and mitochondrial mass in v79 cells (Xu et al., 2014). After mice were subjected to total-body ^60^Co γ-irradiation, treatment with AMF markedly extends average survival time, alleviates impairment of the hematopoietic system and promotes its recovery (Qu et al., 2019). Furthermore, treatment with AMF attenuates radiation-induced oxidative stress through the increase of the SOD activity and GSH level (Qu et al., 2019). In addition, AMF significantly increases the expression of TNFAIP2 (Qu et al., 2019), which plays a role in Wnt/β-catenin and NF-κB signaling pathways (Chen et al., 2014).

### 3.7 Metabolism Regulation

Metabolic disorders such as type 2 diabetes mellitus (T2DM) and metabolic syndrome (MS) are prevalent worldwide and are associated with the disruption of glucose and lipid metabolism (Cho et al., 2018). The changes of general metabolic parameters involve in insulin level, fat mass, body weight and glucose tolerance (Qin et al., 2018). T2DM is characterized by increasing circulating glucose associated with abnormalities in carbohydrate, protein and fat metabolism caused by insufficiency of insulin secretion and insulin resistance (Alfa and Kim, 2016). The major characteristics of MS, as a collection of metabolic abnormalities, include cardiovascular dysfunction, hyperglycemia, hypertension, dyslipidaemia, insulin resistance, abdominal obesity and fatty liver (Torris et al., 2014).

It is reported that AMF could inhibit protein tyrosine phosphatase 1B (PTP1B) activity, therefore AMF has been proposed as a strategy for the treatment of T2D and obesity (Na et al., 2007). AMF treatment increases the phosphorylation of insulin receptor (IR) which is essential for the insulin signaling cascade in 32D cells with high-expressing IR. These results demonstrate that AMF enhances the activation of insulin signaling through inhibiting PTP1B activity (Na et al., 2007). Su et al. (2019) reveal that AMF ameliorates the glucose and lipid metabolism disorder, the hepatic lipid accumulation of hepatic steatosis and repairing the histomorphologic change of pancreas. The abnormality of insulin signaling pathway plays an important role in the development of diabetes, so it is important to study the insulin signaling pathway (Brazil and Hemmings, 2001). PI3K/Akt pathway is the key mediator in the metabolic function of insulin (Yao et al., 2014). Through activating the PI3K/Akt pathway, AMF exerts anti-diabetic effects by regulating the activities of key enzymes in glucose and lipid metabolism, increasing the insulin secretion and improving the insulin signal transduction (Su et al., 2019). Zhang et al. (2019) reveal that AMF plays a pivotal role in the treatment of T2D by reducing inflammatory responses, lowering blood lipids, activating the PPARγ and PI3K/Akt signaling pathway in the KKAy insulin-resistant diabetes mice. Qin et al. (2018) show that AMF protects against cardiovascular ardiovascular and liver dysfunction by involving the modulation of Ang II signaling and oxidative stress through the regulation of NADPH oxidase in high fructose and fat diet (HFFD)-induced MS rats. AMF protects against cardiovascular dysfunction by increasing fractional shortening and decreasing systolic blood pressure, estimated LV mass, LVIDd, relative wall thickness, LVPWd, cardiac stiffness and LV wet weight (Qin et al., 2018). AMF also protects against liver dysfunction through increasing GSH, SOD level and CAT activities, and decreasing NADPH oxidase activities (Qin et al., 2018). In addition, Chen et al. (2016) demonstrate that AMF can protect against high fat diet-induced metabolic dysfunction and inhibit 3T3-L1 adipocyte differentiation. Mechanically, AMF not only promotes ROS generation, but also decreased CCAAT/enhancer-binding protein (C/EBP) *β* expression, and results in the inhibition of mitotic clonal expansion (MCE) (Chen et al., 2016). In summary, AMF inhibits C/EBPα and PPARγ expression, suppresses molecular pathways that responsible for the formation of lipid droplets, and leads to the inhibition of early and terminal differentiation (Chen et al., 2016).

### 3.8 Anxiolytic/Antidepressant

The anxiolytic effect is studied using the elevated plus maze (EPM), hole-board and light-dark tests (Durcan and Lister, 1989). The tail suspension tests (TST) and forced swimming tests (FST) models are used to evaluate the antidepressant effect (Steru et al., 1985). Ishola *et al* obtains evidences for the anxiolytic/antidepressant effect of AMF in mice, and the results suggest that AMF attenuates anxiety by increasing the time spent on the open arms in the EPM, the number of head-dips in the hole-board test and the exploration of the light chamber in the light-dark test (Ishola et al., 2012). In addition, AMF produces its anxiolytic effect through involving GABAergic (ionotropic GABA receptor) system, while the antidepressant effect through interacting with serotonergic (5-HT2 receptors) and noradrenergic (α1-and α2-adrenoceptors) systems (Ishola et al., 2012).

### 3.9 Anti-cancer Effect

**FIGURE 2:**
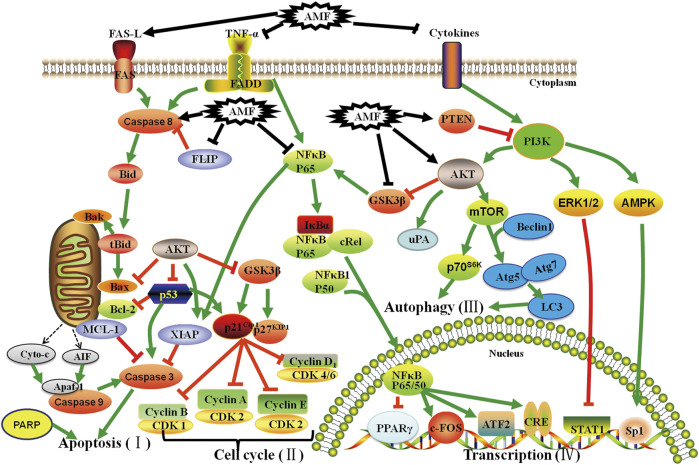
Effect of AMF on Apoptosis (I), Cell cycle (II), Autophagy (III) and Transcription (IV) of various cancers through different molecular signaling pathways. AMF: Amentoflavone; T: Inhibition; ↑: Activation; T: Inhibition by AMF; ↑: Activation by AMF.

**FIGURE 3:**
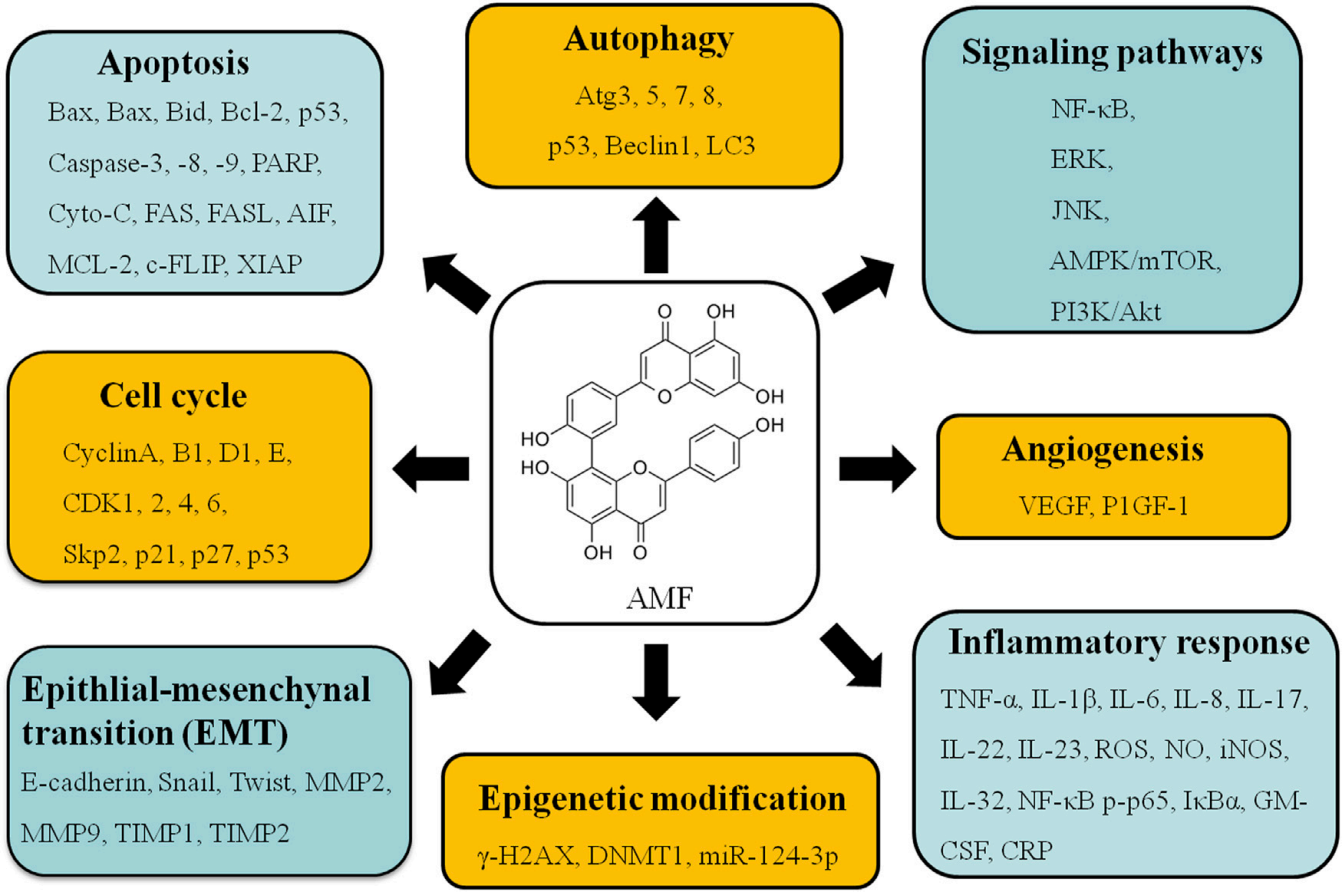
Various molecular targets and signaling regulation modulated by AMF treatment.

Increasing evidences demonstrate that AMF controls cell proliferation, apoptosis, invasion, metastasis, autophagy, transcription and drug-resistance in various types of cancers, such as lung cancer (Banerjee et al., 2002a; Banerjee et al., 2002b; Jung et al., 2017; Hu et al., 2018; Park and Kim, 2019; Shen et al., 2019; Kim et al., 2020; Chen et al., 2021), cervical cancer (Lee et al., 2011), ovarian cancer (Liu et al., 2017a; Zhang et al., 2020), bladder cancer (Chiang et al., 2019), osteosarcoma (Pan et al., 2017; Lee et al., 2019), melanoma (Guruvayoorappan and Kuttan, 2007; 2008b; a; Siveen and Kuttan, 2011), breast cancer (Lee et al., 2009; Pei et al., 2012; Lee et al., 2013; Chen et al., 2015; Aliyev et al., 2021), liver cancer (Zheng et al., 2016; Chen et al., 2017a; Lee et al., 2018a; Lee et al., 2018b; Tsai et al., 2018), brain cancer (Yen et al., 2018; Zhaohui et al., 2018; Hsu et al., 2019; Chen et al., 2020c), and oral squamous cell carcinoma (Chen et al., 2020b) *via* regulating kinds of signaling pathways (Figure 2). These studies provide a lot of evidences that AMF is a potential effective multi-targeting drug for the prevention and treatment of a variety of cancers. AMF has a series of molecular targets and the underlying mechanisms are mainly through regulating the expression of different genes involved in cancer cell growth, cell cycle, apoptosis, autophagy, metastasis, angiogenesis, and epigenetic modification, etc (Table 2 and Figure 3).

**TABLE 2:** AMF and the underlying mechanisms against different cancers.

Cancer	Models	Biological activities	Molecular mechanisms	References
Lung cancer	A549 cells	PGE2 biosynthesis suppression	COX-2/iNOS↓	Banerjee et al. (2002b)
	TNF-α-activated A549 cells	inhibition of NF-κB/DNA binding activity	COX-2↓, IκBα↓, PPAR-γ↑	Banerjee et al. (2002a)
	A549 cells	a potential PARP-1 inhibitor, Cytotoxic of carboplatin↑	PARP-1↓	Hu et al. (2018)
	A549 and WI-38 cells	induction of autophagy	Atg7↑, Beclin1↑, Atg3↑, LC3↑, p53↑, p-P21↑, SIRT1↑	Park and Kim, (2019)
	H1299 and H358 cells	anti-growth and pro-apoptotic activities	CyclinD1↓, CDK4↓, CDK6↓, Caspase3↑, Bax↑, Bcl2↓, CIP2A↓	Shen et al. (2019)
	TGF-β-induced A549 cells	anti-metastatic activity	E-cadherin↑, Snail↓, Twist↓	Kim et al. (2020)
	CL-1-5-F4 cells	Cell-cycle arrest, apoptosis induction, NF-κB signaling inhibition, growth and invasion inhibition	P27↑, Cleaved-caspase3↑, Cleaved-caspase8↑, MMP2↓, MMP9↓, CyclinD1↓, VEGF↓	Chen et al. (2021)
	A549 cells, NCI-H460 cells, A549 tumor xenograft mice	Inhibit cell proliferation	AKR1B10↓	Jung et al. (2017)
Cervical cancer	SiHa and CaSki cells	apoptosis induction, cell cycle arrest at sub-G1 phase	P53↑, P21↑, P27↑, Cyclin E↓, Cyclin A↓, p-pRb↓, PPAR-γ↑, PTEN↑, COX-2↓, IL-32↓, Bcl2↓, Bax↑, Caspase3↑, Caspase9↑, E7↓	Lee et al. (2011)
Ovarian cancer	SKOV3 and OVCAR-3 cells	cell apoptosis and cell cycle arrest induction	Skp2↓, P21↑, P27↑, CDK2↓, ROS/AMPK/mTOR signaling↑	Liu et al. (2017a)
	SKOV3 cells	cell cycle G2/M arrest, DNA damage induction	P21↑, CDK1/2↓, γ-H2AX↑, Rad51↑	Zhang et al. (2020)
Bladder cancer	TSGH8301	apoptosis induction, inhibition of anti-apoptotic and metastasis-associated proteins	FAS↑, FASL↑, Bax↑, MCL-1↓, C-FLIP↓, MMP2↓, MMP9↓, VEGF↓, uPA↓, CyclinD1↓	Chiang et al. (2019)
Osteosarcoma	U2OS cells	inhibition of metastasis-associated proteins, cell migration, and cell invasion	p-ERK↓, NF-κB activity↓, MMP2↓, MMP9↓, VEGF↓, uPA↓	Pan et al. (2017)
	U2OS cells	Tumor progression inhibition	p-ERK↓, NF-κB p-P65↓, XIAP↓, MMP9↓, VEGF↓, CyclinD1↓	Lee et al. (2019)
Melanoma	B16F-10-injected-C57Bl/6 mice	Inhibition of pulmonary metastasis	TIMP1↑, TIMP2↑, IL-6↓, IL-1β↓, GM-CSF↓, TNF-α↓, NF-κB ↓, c-FOS↓, ATF2↓, CRE-B↓	Guruvayoorappan and Kuttan, (2007)
	B16F-10-injected-C57Bl/6 mice	attenuation of tumor invasion, proliferation and angiogenesis	MMP2↓, MMP9↓, Prolyl hydroxylase↓, lysyl oxidase↓, VEGF↓, ERK1/2↓, IL-6↓, TNF-α↓, nm23↑, GM-CSF↓, IL-1β↓, STAT-1↑	Guruvayoorappan and Kuttan, (2008a)
	B16F-10 cells	apoptosis induction	NO↓, IL-6↓, TNF-α↓, Bcl2↓, GM-CSF↓, IL-1β↓, P53↑, Caspase3↑	Guruvayoorappan and Kuttan, (2008b)
	B16F-10 cells	apoptosis induction, cell G0/G1 phase arrest	P21↑, P27↑, Bax↑, Caspase9↑, CyclinD1↓, Bid↓, Bcl2↓, Caspase9↑, P53↑	Siveen and Kuttan, (2011)
Breast cancer	SKBR3 cells	blockade of fatty acid synthesis, apoptosis induction, anti-proliferation	Cleaved-caspase3↑, PARP↑, FASN activity↓, DNA fragmentation↑	Lee et al. (2009)
	MCF-7 cells	apoptosis induction, cell cycle arrest	ROS↓, Bcl2↓, Bax↑, AIF↑, P53↑, Bid↓, Caspase3↑	Pei et al. (2012)
	SKBR3 cells	fatty acid synthase inhibition, enhance chemo-preventive or chemotherapeutic activity	FASN↓, HER2↓, PEA3↑, PARP↑, SREBP-1↓, Caspase3↑, p-AKT↓, p-JNK↓, p-mTOR↓	Lee et al. (2013)
	MCF-7 cells	anti-angiogenesis and anti-metastasis induction	VEGF↓, MMP2↓, MMP9↓, NF-κB p-P65↓	Chen et al. (2015)
	MCF-7 cells, MDA-MB-231 cells, MCF-10A cells	Aromatase inhibition, cytotoxic, bind to the active site of hCYP19A1	hCYP19A1↓	Aliyev et al. (2021)
Hepatocellular carcinoma	HepG2 cells	Improvement of insulin resistance	PI3K↑, AKT↑, p-AKT↑, GCK↑, PFK-1↑, TNF-α↓, PK↑, GSK-3↓, PEPCK↓, IL-6↓, G-6-Pase↓, IL-8↓, CRP↓	Zheng et al. (2016)
	Sorafenib-resistant Sk-Hep1 cells	enhance sorafenib-induced cytotoxicity, trigger sorafenib-induced apoptosis	DNA fragmentation↑, XIAP↓, MCL-1↓, C-FLIP↓, Cleaved-caspase3↑, Cleaved-caspase8↑, Cyto-c↑	Chen et al. (2017a)
	SK-Hep1 tumor-bearing mice	apoptosis induction, enhance sorafenib-inhibited tumor growth	XIAP↓, MCL-1↓, C-FLIP↓, p-AKT↓, Caspase9↑, Caspase8↑, Caspase3↑, p-ERK↓	Tsai et al. (2018)
	SK-Hep1 cells	Reduction of cell viability, NF-κB activation, and cell invasion	p-ERK↓, MMP9↓, XIAP↓, VEGF↓, CyclinD1↓	Lee et al. (2018b)
	SK-Hep1 tumor-bearing mice	Inhibition of tumor growth and ERK/NF-κB activation	p-ERK↓, MMP9↓, XIAP↓, MCL-1↓, C-FLIP↓, VEGF↓, CyclinD1↓, NF-κB p-P65↓	Lee et al. (2018a)
Brain cancer	U87MG Cells	apoptosis induction, inhibition of NF-κB-modulated anti-apoptotic signaling	NF-κB activity↓, MCL-1↓, C-FLIP↓	Yen et al. (2018)
	U87, LV229, U251, LN18 and U373 cells	proliferation inhibition, apoptosis induction, glycolysis suppression	ROS/AMPK↑, Sp1↑, DNMT1↓, miR-124-3p↑	Zhaohui et al. (2018)
	GBM8401	blockage of ERK/NF-κB signaling, inhibition of tumor growth	ERK/NF-κB activity↓, MMP2↓, MMP9↓, XIAP↓, CyclinD1↓, VEGF↓	Hsu et al. (2019)
	U251 and U373 cells	cell proliferation suppression, cell death induction, triggering autophagy-dependent ferroptosis	MDA↑, GSH↓, LC3B↑, Beclin1↑, ATG5↑, ATG7↑, FTH↓, lipid OS↑, CyclinD1, CyclinB1↓, CDK2↓, CDK4↓, p-AMPK↓, p-mTOR↓, p-P70↓	Chen et al. (2020c)
Oral Squamous Cell Carcinoma	SAS cells	Increasing cisplatin-induced cytotoxicity, enhancing cisplatin-induced apoptosis, augmenting cisplatin-suppressed invasion and migration ability	NF-κB p-P65↓, Cleaved caspase3↑,Bax↑, BAK↑, Cleaved caspase8↑, Cleaved caspase9↑	Chen et al. (2020b)

#### 3.9.1 Cell Cycle Arrest

AMF has been confirmed to induce cell cycle arrest in multiple cancer cells, such as, lung (Shen et al., 2019), cervical (Lee et al., 2011), melanoma (Siveen and Kuttan, 2011), and ovarian cancer cells (Liu et al., 2017a). In non-small cell lung cancer cells, AMF treatment significantly increases the cell population at G1/G0 phase by decreasing the expression of cyclin D1, CDK4 and CDK6 in both H358 and H1299 cells (Shen et al., 2019). Similarly, AMF treatment induces a significant cell cycle arrest at G1/G0 phase via elevating the levels of p21 and p27 and decreasing the level of CDK2 in SKOV3 and OVCAR-3 cells (Liu et al., 2017a). Treatment of B16F-10 cells with AMF could also increase the percentage of cells in the sub-G0/G1 phase by downregulating cyclin D1 and Bid proteins (Siveen and Kuttan, 2011). Additionally, the treatment of SiHa and CaSki cells with AMF induces cell cycle arrest at the sub-G1 phase through the down-regulation of p-pRb and G1/S cyclins and the up-regulation of p21 and p27 *via* a p53-dependent pathway (Lee et al., 2011). Besides the effect of AMF on G1-phase cell cycle arrest, AMF treatment can inhibit cell proliferation, interrupt the balance of microtubule dynamics and arrest cells at the G2 phase *via* increasing p21 expression and decreasing CDK1/2 expression in ovarian cancer SKOV3 cells (Zhang et al., 2020).

#### 3.9.2 Apoptosis Induction

Apoptosis is the process of programmed cell death. The induction of cell apoptosis is an important strategy for anti-cancer activity (Taylor et al., 2008). Caspase activation plays a crucial role in apoptosis-mediated cancer cell death (Fischer et al., 2007). Caspase-3 mediates the proteolytic cleavage of poly adenosine diphosphate-ribose polymerase (PARP) and plays an important role in condensation and degradation of chromatin in cells. A large number of reports reveal the effect of AMF in the induction of apoptosis through either intrinsic (mitochondria-mediated) and/or extrinsic pathway in different cancer cells. In the mitochondria-mediated pathway, AMF treatment decreases the expression of anti-apoptotic factor Bcl-2 and increases the expression of pro-apoptotic factor Bax, thereby cytochrome-C is released to cytosol accompanying the activation of caspases-3/-9 and PARP in cervical cancer SiHa and CaSki cells (Lee et al., 2011). Additionally, AMF induces MCF-7 cells to undergo apoptosis via the ROS- and Ca^+2^-involved mitochondria-dependent pathway (Pei et al., 2012). In B16F-10 melanoma cells, AMF treatment induced apoptosis through p53-dependent intrinsic apoptotic pathway by increasing Bax and caspase-9 protein levels (Siveen and Kuttan, 2011). In addition to the intrinsic pathway, there are some reports on the apoptotic effect of AMF through the extrinsic pathways. AMF inhibits multiple anti-apoptotic proteins, such as XIAP, C-FLIP and Mcl-1 (Igney and Krammer, 2002). In SK-Hep1R cells, AMF not only promotes sorafenib-induced apoptosis through intrinsic pathway via enhancing cleaved-caspase-8/3 and cyto-c release, but also promotes sorafenib-induced extrinsic apoptosis pathway through inhibiting the expression of XIAP, C-FLIP and Mcl-1 proteins (Chen et al., 2017a). In bladder cancer, AMF induces FAS/FASL-dependent extrinsic apoptosis through increasing pro-apoptotic protein levels of FAS and FASL (Chiang et al., 2019). Moreover, AMF also induces the apoptotic pathway by increasing the expressions of PTEN (Lee et al., 2011), phosphorylated JNK (Lee et al., 2013) and decreasing the expressions of phosphorylated AKT (Tsai et al., 2018) and ERK (Lee et al., 2019).

#### 3.9.3 Autophagy Induction

Autophagy is a cell degradation pathway used to remove damaged or redundant proteins and organelles, and is also associated with tumorigenesis (Mathew et al., 2007). Mammalian target of rapamycin (mTOR) is one part of mTOR complex 1 (mTORC1) and a major regulator of cell growth and autophagy (Jewell et al., 2013). ATG, Beclin 1 and LC3 are the proteins involved in multiple processes of autophagosome formation and are essential for autophagy (Park and Kim, 2019; Wang and Wang, 2019). Previous studies have confirmed that AMF can induce autophagic cell death in several cancer cells, such as glioma (Chen et al., 2020c) and lung (Park and Kim, 2019). AMF increases the autophagic flux of glioma U251 and U373 cells via up-regulating the autophagy-relevant proteins, such as Beclin1, LC3B, ATG5, ATG7 (Chen et al., 2020c) and the phosphorylation of AMPK or suppressing the phosphorylation of mTOR and p70S6K (Chen et al., 2020c). Moreover, AMF promotes ferroptosis in autophagy-dependent manner. The knockdowns of ATG7 and autophagy inhibitor Baf A1 are able to abrogate AMF-inducing ferroptosis and autophagic cell death in glioma cells (Chen et al., 2020c).

#### 3.9.4 Signaling Pathways Regulation

Previous studies have confirmed that AMF exerts an inhibitory effect on multiple signaling pathways, such as NF-κB, PI3K/AKT, ERK, JNK and AMPK/mTOR pathway. As a heterodimeric transcription factor, NF-κB is composed of p50 and p65 subunits, mediates tumor invasion and metastasis through regulating the expressions of metastasis-associated proteins such as XIAP, MMP-2, MMP-9, cyclinD1, and VEGF (Rasmi et al., 2020). *In vitro* studies, AMF suppresses cell viability, invasion and migration of different types of cancers, including glioblastoma (Hsu et al., 2019) and HCC (Lee et al., 2018b) through inhibiting NF-κB activation and NF-κB-mediated downstream gene expression. Similarly, AMF reduces the invasion ability of NSCLC cells through blocking NF-κB signaling pathway and NF-κB p65 nuclear translocation (Chen et al., 2021). Furthermore, AMF inhibits osteosarcoma and HCC progression *in vivo* by suppressing ERK/NF-κB activation (Lee et al., 2018a; Lee et al., 2019). AMF also enhances insulin resistance of HepG2 cells through the PI3K-Akt signaling pathway (Zheng et al., 2016). In addition, AMF induces caspase-dependent apoptosis, exerts FASN-inhibitory activity and decreases cell proliferation via suppressing HER2 activation and modulating the expressions of Akt, mTOR and p-JNK in SKBR3 cells (Lee et al., 2009; Lee et al., 2013). AMF represses ovarian cancer and the expression of Skp2 through ROS/AMPK/mTOR signaling pathway in xenograft mouse model (Liu et al., 2017a). AMF inhibits cell growth and induces ferroptosisin in glioma U251 and U373 cells through modulating iron homeostasis via repressing ferritin heavy chain (FTH). AMF suppresses FTH expression through the induction of autophagy *via* AMPK/mTOR/p70S6K signaling pathway (Chen et al., 2020c).

#### 3.9.5 Metastasis and Angiogenesis

Epithelial mesenchymal transition (EMT) is essential for driving plasticity during development, and is believed to play an important role in the metastasis of many cancers (Jou and Diehl, 2010; De Craene and Berx, 2013). Several proteins and transcription factors, such as Ecadherin, Snail and Twist, have been proved to drive EMT process (Kalluri and Weinberg, 2009). AMF inhibits EMT via the inhibition of Snail1/Twist signaling axis in both A549 and HT29 cells (Kim et al., 2020). MMP-2 and MMP-9 promote the degradation of basement membrane and lead to tumor cell invasion and metastasis (Liu et al., 2017b). AMF prevents bladder cancer invasion and migration by reversing EMT via NF-κB inactivation and by reducing the expression of MMP-2, MMP-9 and uPA (Chiang et al., 2019).

Angiogenesis is critical for multiple physiological and pathological processes (Guruvayoorappan and Kuttan, 2008c). Angiogenesis is a mandatory factor for tumor metastasis. The inhibition of angiogenesis is a strategy for tumor treatment (Liu et al., 2017b). *In vitro* studies, AMF may induce anti-angiogenesis of MCF cells via inhibiting the expression and secretion of VEGF through NF-κB inactivation (Chen et al., 2015). AMF also attenuates tumor invasion and angiogenesis in osteosarcoma U2OS cells (Pan et al., 2017), melanoma B16F10 cells (Guruvayoorappan and Kuttan, 2008b), and NSCLC cells (Chen et al., 2021). *In vivo* study, AMF treatment reduces B16F-10 melanoma cells-induced lung metastasis in transplanting C57BL/6 mice (Guruvayoorappan and Kuttan, 2007; 2008a). It is reported that AMF can inhibit VEGFA-induced chorioallantoic membrane neovascularization in xenograft colon carcinoma mice. AMF inhibits endothelial cell migration and VEGFA or PIGF-1-induced capillary-like tube formation, and prevents the interaction between VEGFs and VEGF receptor 1/2 (VEGFR-1/-2) by binding with proangiogenic VEGFs (Tarallo et al., 2011).

In addition to the anti-cancer effect of AMF by inhibiting angiogenesis, AMF also plays an important role in some non-neoplastic diseases. In hypertrophic scar fibroblasts, AMF inhibits angiogenesis of endothelial cells by inhibiting the viability, migration and tube formation (Zhang et al., 2014). In vasodilation, AMF relaxes vascular smooth muscle via the activation of endothelium-dependent NO-cGMP signaling pathway which may be involved in the functions of K^+^ and Ca^2+^channels (Kang et al., 2004). It is reported that AMF may exert a vasodilating effect through a NO-independent, cGMP-PDE5-dependent mechanism in the smooth muscle cells of the arterial wall (Dell'Agli et al., 2006).

#### 3.9.6 Epigenetic Modification

Epigenetic modification of nucleic acids occurs broadly both in DNA and in RNA and is involved in growth, heredity and diseases (Chen et al., 2017b). Previous studies reveal that AMF exerts anticancer effects via regulating the expression of epigenetic modification genes in cancer cells. AMF attributes to apoptosis and glycolysis inhibition by up-regulating miR-124-3p through repressing DNMT1. Followed that, AMF suppresses DNMT1 expression via the activation of ROS/AMPK and Sp1 signaling pathways (Zhaohui et al., 2018). Moreover, in ovarian cancer cells AMF enhances the occurrence of DNA damage by increasing the expression levels of γ-H2AX and Rad51 (Zhang et al., 2020).

#### 3.9.7 Drug Resistance

Drug resistance remains the main limiting factor for the cure of cancer patients (Vasan et al., 2019). Some traditional Chinese medicines are becoming new strategies for tumor treatment by combining chemotherapeutic drugs. AMF can synergistically increase the cytotoxic effects of carboplatin in A549 cells and may be a potential chemosensitizer to carboplatin for NSCLC through PARP-1 *in vitro* and *in vivo* (Hu et al., 2018). AMF not only significantly enhances cisplatin-induced cytotoxicity *via* NF-κB inactivation, but also significantly increases the cisplatin-mediated inhibition of cell proliferation, invasion and migration of oral squamous carcinoma SAS cells (Chen et al., 2020b). Moreover, AMF enhances insulin resistance in HepG2 cells and the underlying mechanisms may be involved in inflammatory cytokine expression, the processes of glucose oxygenolysis, gluconeogenesis, glycogen synthesis and the PI3K-Akt signaling pathway (Zheng et al., 2016).

## 4 The Toxicity or Undesirable Effects of Amentoflavone

In addition to the extensive studies on the pharmacological effects, the toxicity or undesirable effects of AMF are also reported (Table 3). Cytochrome P450 enzymes (CYPs) are the typical drug-metabolizing enzymes (phase I metabolism). CYP enzymes are responsible for the breakdown of xenobiotics and endogenous components, such as environmental compounds and drugs, into metabolites (Kimura et al., 2010). Several studies have reported that the interaction of AMF with drugs inhibits the catalytic activities of CYP enzymes (von Moltke et al., 2004; Chaudhary and Willett, 2006; Kimura et al., 2010; Park et al., 2020). It is reported that AMF is a highly potent inhibitor of CYP2C9 with an IC_50_ value of 0.035 μM, and also inhibites CYP2C19, CYP 2D6 and CYP 3A with IC_50_ values of 23.6, 24.3, 4.8 μM, respectively (von Moltke et al., 2004). The calculated IC_50_ for CYP1A1 (38 ± 19 μM) by AMF is higher than the calculated IC_50_ for CYP1B1 (4.6 ± 1.4 μM) through regression curves plotting percent EROD inhibition. AMF inhibits CYP1A1with *Ki* value of 1.6 ± 0.78 μM in uncompetitive manner and CYP1B1 with *Ki* value of 0.99 ± 0.31 μM in competitive manner by EROD activity assay (Chaudhary and Willett, 2006). AMF displays a competitive-non-competitive mixed type of inhibition on CYP2C9 or CYP3A4 by Lineweaver-Burk plot analysis with IC_50_ values of 0.03 and 0.07 μM, respectively. The Lineweaver-Burk plots, secondary reciprocal plots and Dixon plots researches in human liver microsomes (HLMs) reveal that AMF strongly inhibits CYP1A2, CYP2A6, CYP2B6, CYP2C8, CYP2C9, CYP2C19, CYP2D6, CYP2E1 and CYP3A activity with IC_50_ values of 4.4, 11.9, 7.1, 0.084, 0.15, 3.4, 2.6, 3.3 and 1.3 μM, respectively. AMF inhibits CYP2C8-mediated amodiaquine N-deethylation activity with *Ki* value of 0.083 μM in noncompetitive-dependent manner (Park et al., 2020).

**TABLE 3:** The inhibitory effects of AMF on different enzymes (targets).

Substrate	Enzyme (target) source	IC50 (μM)	Ki (μM)	Refences
TBA	Microsomal lipid peroxidation	74.1 ± 0.8		Cholbi et al. (1991)
IP_t_	pLCr1	29		Lee et al. (1996)
cAMP	Phosphodiesterase (PDE)	0.27		Saponara and Bosisio, (1998)
	COX-1	12.4		Bucar et al. (1998)
Flurbiprofen	CYP29C	0.035		von Moltke et al. (2004)
S-Mephenytoin	CYP2C19	23.6		
Dextromethorphan	CYP2D6	24.3		
Triazolam	CYP3A	4.8		
	Cathepsin B	1.75		Pan et al. (2005)
EROD	CYP1A1	38 ± 19	1.6 ± 0.78	Chaudhary and Willett, (2006)
	CYP1B1	4.6 ± 1.4	0.99 ± 0.31	
Insulin receptor	PTP1B	7.3	5.2	Na et al. (2007)
	β-secretase (BACE-1)	1.54		Sasaki et al. (2010)
Diclofenac	CYP2C9	0.03	0.007	Kimura et al. (2010)
Testosterone	CYP3A4	0.07	0.027	
	JAK2	5		Ma et al. (2014)
4-MU-O-glucuronidation	UGT1A1	0.78 ± 0.19	2.21 ± 1.14	Lv et al. (2018)
	UGT1A3	2.55 ± 0.07	0.73 ± 0.31	
	UGT1A6	3.43 ± 0.83	4.05 ± 0.21	
	UGT1A7	0.12 ± 0.02	0.29 ± 0.03	
	UGT1A8	1.72 ± 0.54	0.85 ± 0.15	
	UGT1A9	4.54 ± 0.63	0.46 ± 0.12	
	UGT1A10	2.71 ± 0.43	3.45 ± 0.59	
	UGT2B4	7.06 ± 0.82	5.18 ± 2.06	
	UGT2B7	15.91 ± 4.85	11.51 ± 5.24	
	UGT2B15	16.86 ± 5.67	9.88 ± 0.94	
	UGT2B17	2.13 ± 0.23	2.16 ± 1.57	
6-CF	OAT3	2		Qiao et al. (2019)
Phenacetin	CYP1A2	4.4	3.1 ± 0.6	Park et al. (2020)
Coumarin	CYP2A6	11.9		
Bupropion	CYP2B6	7.1	7.9 ± 1.1	
Amodiaquine	CYP2C8	0.084	0.018 ± 0.002	
Diclofenac	CYP2C9	0.15	0.032 ± 0.007	
Omeprazole	CYP2C19	3.4		
Dextromethorphan	CYP2D6	2.6		
Chlorzoxazone	CYP2E1	3.3		
Midazolam	CYP3A	1.3	4.5 ± 0.5	
DDAOG	β-glucuronidase	0.62	0.24	Tian et al. (2021)
SN38G		0.49	1.25	

UDP-glucuronosyl transferases (UGTs), the most important class of detoxification enzymes, are known as human phase II drug metabolizing enzymes (Lv et al., 2018). UGTs play key roles in the detoxification and metabolic elimination of a wide variety of endogenous compounds. The effects of AMF on UGTs (including UGT1A1, UGT1A3, UGT1A4, UGT1A6, UGT1A7, UGT1A8, UGT1A9, UGT1A10, UGT2B4, and UGT2B17) are carefully revealed that the IC_50_ values and *Kis* of AMF against various human UGTs with ranging from 0.12 to 16.81 μM, 0.29 to 11.51 μM, respectively. In addition, AMF is a noncompetitive inhibitor of UGT1A1 mediated NCHN-O-glucuronidation, a competitive inhibitor of UGT1A4 mediated TFP-N-glucuronidation, a competitive inhibitor of UGT1A1 mediated 4-MU-O-glucuronidation and a competitive inhibitor of UGT1A9 mediated propofol or 4-MU-O-glucuronidation (Lv et al., 2018).

Besides those, Chiolbi et al. (1991) investigate that AMF can act at the initiation stage of CCl4-induced rat liver microsomal lipid peroxidation by interfering with the metabolism of CCl4. AMF is a potent inhibitor of TBA-reactive material formation with IC_50_ value of 74.1 ± 0.8 μM (Cholbi et al., 1991). Lee et al. (1996) reveal that AMF inhibits the PLCy1 activity with an IC_50_ of 29 μM and also reduces intracellular total inositol phosphates (lPt) in PDGF-treated NIH3T3y1 cells with an IC50 of 9.2 μM. Lipolysis in fat cells is regulated by cAMP synthesis which is stimulated by adenylate cyclase activation or the reduction of cAMP destruction by phosphodiesterase (PDE) inhibition. Saponara and Bosisio (1998) demonstrate that AMF is a potent inhibitor on adipocyte-derived PDE with the IC_50_ value of 0.27 μM in rat adipose tissue. AMF is proved to be a selective inhibitor of cyclooxygenase (COX)-1 catalysed prostaglandin biosynthesis with an IC_50_ value of 12.4 μM *in vitro* (Bucar et al., 1998). Cathepsin B (CatB), a lysosomal cysteine protease, plays roles in intracellular protein catabolism and in other physiological processes (e.g., hormone activation, processing of antigens in the immune response and bone turnover) (Pan et al., 2005). Pan et al. (2005) report that AMF has a strong inhibitory activity against human CatB with a IC_50_ value of 1.75 μM. Inhibition of protein tyrosine phosphatase 1B (PTP1B) has been proposed as a strategy for the treatment of type 2 diabetes and obesity (Na et al., 2007). Na et al. (2007) suggest that AMF inhibits PTP1B with an IC_50_ value of 7.3 ± 0.5 μM and is a non-competitive inhibitor with a *Ki* value of 5.2 μM by Kinetic study. Moreover, AMF shows strong inhibitory activity against *β*-secretase (BACE-1) with IC_50_ values of 1.54 μM and can result in accumulation and deposition of amyloid *β* (A*β*) peptides in Alzheimer’s disease (Sasaki et al., 2010). AMF inhibits JAK2 activity in a dose-dependent manner with an IC_50_ value of 5 μM (Ma et al., 2014). AMF also shows strong inhibition on OAT3, a member of the solute carrier family of membrane transporters, with an IC_50_ of 2.0 μM (Qiao et al., 2019). *β*-glucuronidase (GUS) plays a pivotal role in the metabolism and reactivation of a vast of glucuronide conjugates of both endogenous and xenobiotic compounds (Tian et al., 2021). AMF inhibits GUS-mediated SN38G and DDAOG hydrolysis with the IC_50_ values of 0.49 and 0.62 μM, respectively. AMF is a competitive type inhibitor for GUS-mediated SN38G hydrolysis and displays a mixed type inhibition against GUS-mediated DDAOG hydrolysis with the *Ki* values of 1.25 and 0.24 μM by inhibition kinetics studies, respectively (Tian et al., 2021).

## 5 Molecular Docking Simulation of Amentoflavone Through *in silico* Approach

Molecular docking and molecular dynamics simulation are algorithm-based virtual screening methods searching for candidate drugs or molecules in a short time and serving for experimental studies (Alonso et al., 2006; De Vivo et al., 2016; Wang and Zhu, 2016). As a potential molecule with the activities of anti-inflammation (i.e., p38 MAPK signaling pathway) (Kadam et al., 2007), anti-tubercular (i.e., tuberculosis) (Nayak et al., 2018; Kumar et al., 2019), anti-chagas (Marinho et al., 2021) and anti-virus (i.e., SARS-CoV-2) (Ghosh et al., 2020; Lokhande et al., 2020), AMF is virtually screened through molecular docking and molecular dynamics simulation of *in silico* approaches in recent researches (Table 4).

**TABLE 4:** Molecular docking proteins of AMF through *in silico* study.

Proteins	Binging energy (Kcal/mol)	Interacting residues	References
P38 MAPK	−26.34	Val30, Tyr35, Met109, Glu71, Arg173, Lys53	Kadam et al. (2007)
UGM	−10.4	Glu143, Phe157, Trp166, Asn177, Asn282	Nayak et al. (2018)
Ask	−9.9	Thr156, Leu214, Leu212, Ala205, Arg355	(Kumar et al., 2019)
DdlA	−10.7	Lys194, Asn329, Arg316, Glu23, Ser201	
PanC	−10.7	Gly46, Lys160, His44, Asn69	
RplW	−7.4	Ile49, Asp94	
TrpB	−9.7	Arg155, Ala126, Asp319, His129, Thr204, Gly248, Gly247	
Cruzain	−8.0	Gln159, Gln19, Leu160, Met145, Asp161, Gln21, His162, Gly20, Met68, Gly163, Trp26, Gly65, Ala138, Ser64, Cys25, Gly23, Trp184	Marinho et al. (2021)
SARS Cov-3-Chymotrypsin-like protease (3CLpro)	−11.42	Leu141, His163, Gln189, Gln192, Val186	Ryu et al. (2010)
SARS Cov-2-3-Chymotrypsin-like protease (3CLpro)	−9.4	His41, Arg188, Cys44, Met49, Phe140, Asn142, Leu141, Val186, Cys145, Met165, Asp187, Glu166, Gln189	Swargiary et al. (2020)
SARS Cov-2-main protease (Mpro)	−9.2	Thr26, Glu166, Thr25, Tyr54, His172, Leu27, Leu42, Arg188, Asn142, Gly143, Ser144, His164, Leu167, Pro168, His163, Phe140, Cys145, Leu141, Asp187, Gln189, Met165, His41	Ghosh et al. (2020)
SARS Cov-2-main protease (Mpro)	−27.0441	Thr26, Asn142, His163, Glu166	Lokhande et al. (2020)
SARS-CoV-2-main protease (Mpro)	−10.0	Leu141, Thr45, Thr190, Asn142, Glu166, Cys44	Saravanan et al. (2020)
SARS-CoV-2-main protease (Mpro)	−9.7	Glu166, Glu189, Asn142, Ser144, Cys145, Leu141, Gly143	Puttaswamy et al. (2020)
SARS-CoV-2-main protease (Mpro)	−7.589	Glu66, Thr25, His41, Ser46	Patil et al. (2021)
SARS-CoV-2-main protease (Mpro)	−8.1	Asn151, His246	Rameshkumar et al. (2021)
SARS-CoV-2-spike protein	−7.6	Arg457, Ser469, Glu471, Lys458, Asp467	Wei et al. (2020)
SARS-CoV-2-spike protein	−8.7	Gln493, Ser494, Gly496, Gln498, Tyr495, Arg403, Glu493, Asn501, Try453, Tyr505, Leu455, Gly502, Lys417	Puttaswamy et al. (2020)
SARS-CoV-2-spike protein	−8.5	Tyr453, Arg403, Gly496, Asn501, Gln498, Tyr505, Tyr495	Miroshnychenko and Shestopalova, (2021)
SARS-CoV-2-spike protein	−10.2	Val315, Thr319, Thr394, Phe396, Asn628	Rameshkumar et al. (2021)
SARS-CoV-2-RNA-dependent RNA polymerase (RdRp)	−8.1	Ser43, Asp350, Tyr385, Asn394	Rameshkumar et al. (2021)

It is reported that a powerful bond between p38 MAPK signaling pathway and inflammation (Lee et al., 1994). Kadam et al. (2007) explored the potential inhibitory effect of AMF on p38 MAPK using *in silico* study. The docking model predicts that AMF has a more favorable ΔG binding of -26.34 kcal/mol to p38 MAPK than the reported p38 MAPK inhibitor (-17.95 kcal/mol). AMF shows H-bonding which interacts with Met109, Lys53, Glu71, Val30 and Arg173, the carbonyl oxygen of *γ*-Benzopyrone ring which makes *π*-stacking interactions with Tyr35, and *γ*-benzopyrone 2-phenol group which binds to the selectivity pocket by HOMO/LUMO and surface analysis (HD and MESP) (Kadam et al., 2007).

Tuberculosis (TB) has prevailed for millennia and remains a major health problem worldwide (Sabiiti and consortium, 2017). Increasing incidences of multidrug resistant cases of TB are a major threat. AMF is reported to have antibacterial and antitubercular activities (Nayak et al., 2018; Kumar et al., 2019). *In silico* screening, Nayak et al. (2018) and Kumar et al. (2019) identify that AMF can target the drugs of *Mycobacterium tuberculosis* (MTB) and possesses anti-TB activity. *Mycobacterium tuberculosis* uridine diphosphate galactofuranose galactopyranose mutase (UGM) is not only a necessary flavoenzyme for the survival of mycobacteria, but also an important part of cell wall (Nayak et al., 2018). Nayak et al. (2018) find that AMF is a potential effective inhibitor against UGM by virtual screening and interaction analysis. AMF shows a high binding affinity (binding energy of −10.4 kcal/mol) toward UGM and has hydrogen bond interactions with the residues Glu143, Phe157, Trp166, Asn177, Asn282 (Nayak et al., 2018). Meanwhile, Kumar et al. (2019) proclaim that fifteen proteins which are actively involved in molecular function, biological process and cellular component of MTB are shortlisted by virtual screening. Nevertheless, only five drugs of MTB (i.e., Ask, DdlA, PanC, RplW, and TrpB) are inhibited by AMF according to *in silico* analysis (Kumar et al., 2019). AMF inhibits Ask with binding energy of −9.9 kcal/mol by interacting with Leu212, Thr156, Ala205, Leu214, and Arg355 of Ask to form polar contact. The residues Glu23, Ser201, Lys194, Arg316, and Asn329 of DdlA protein can interact with AMF to form polar contact with binding energy of −10.7 kcal/mol (Kumar et al., 2019). Further, AMF interacts with His44, Lys160, Gly46, and Asn69 of PanC protein to form H-bonds with binding energy of −10.7 kcal/mol (Kumar et al., 2019). AMF binds with RplW with an affinity of −7.4 kcal/mol by forming polar contacts with Ile49 and Asp94 residues (Kumar et al., 2019). AMF can also binds with TrpB well with an affinity of −9.7 kcal/mol and forms polar contacts with residues of Gly247, Asp319, Gly248, Ala126, Thr204, His129, and Arg155 residues in protein-ligand complex (Kumar et al., 2019).

Cruzain is a main cysteine protease enzyme of T. cruzi and essential for intracellular parasite replication. It is considered one of the most important targets for new trypanocidal agent development (Avelar et al., 2015). Cruzain has a catalytic site locating at the intersection of two domains, namely *α*-helices and *β*-Sheets, in which the residues are prominent. The molecular docking analysis shows that AMF has an interactive affinity simulations (-8.0 kcal/mol) with the catalytic site of cruzain (Marinho et al., 2021). The interactions between AMF and cruzain are identified. They are three hydrogen bonds with the residues Gly20, Met68 and Ser64, a van der Waals with His162, an Amide-Pi with the Asp161, a Pi-Alkyl with Ala138, and a *π*-*π* stacking with Trp184 (Marinho et al., 2021).

## 6 ANTI-SARS-CoV-2 Effect of Amentoflavone

Coronavirus disease (COVID-19) is an infectious disease caused by severe acute respiratory syndrome coronavirus 2 (SARS-CoV-2). SARS-CoV-2 primarily infects the lungs and causes certain types of pneumonia-like symptoms (Huang et al., 2020; Kumar et al., 2021). COVID-19 is a communicable disease and is spreading internationally. SARS-CoV-2 is a member of coronavirus family and belongs to the beta-coronavirus 2B lineage (Lai et al., 2020). SARS-CoV-2 is composed of four structural proteins [spike (S), membrane (M), envelope (E), nucleocapsid (N) proteins] and sixteen nonstructural proteins (Nsp1−16) (Wang et al., 2020). Spike protein, the most variable structure, is a heavily glycosylated protein and has a receptor binding domain (RBD) (Zhou et al., 2020) which can mediates coronavirus entry into host cells (Bosch et al., 2003; Li, 2016). The main protease (Mpro/3CLpro) in Nsp5 participates in the process of polyproteins which play a critical role in the replication and transcription of SARS-CoV-2 (Kirtipal et al., 2020; Wang et al., 2020). The RNA-dependent RNA polymerase (RdRp) locates in Nsp12 which also participates in the replication/transcription of coronavirus (Kirtipal et al., 2020; Wang et al., 2020). The spike protein mediates SARS-CoV-2 to invade host cells. Moreover, the main protease and RdRp participates in the replication/transcription of SARS-CoV-2 (Kirtipal et al., 2020; Wang et al., 2020). Therefore, the spike protein, main protease, and RdRp are important drug targets of anti-SARSCoV-2.

**FIGURE 4:**
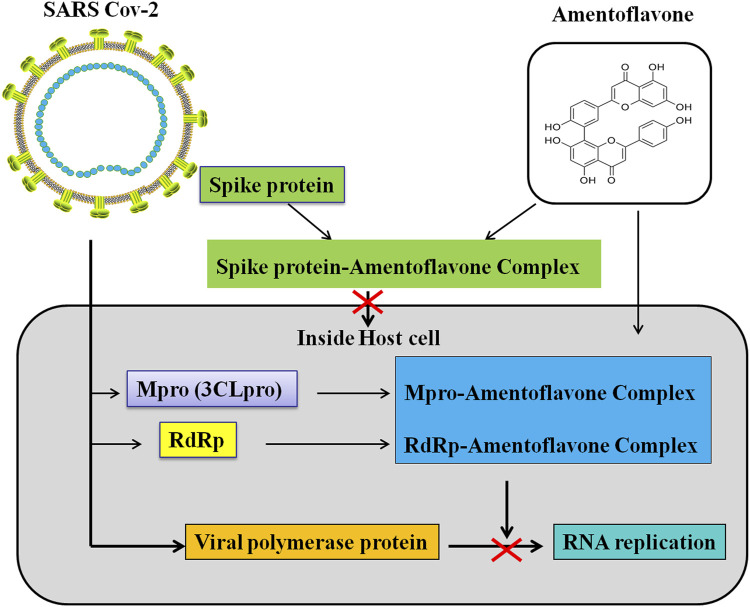
Schematic representation for the functions of AMF in SARS-Cov 2 replication and transcription.

Many previous studies have found that AMF can form a complex with the spike protein, Mpro and RdRp of SARS-CoV-2 (Lokhande et al., 2020; Puttaswamy et al., 2020; Rameshkumar et al., 2021) (Table 4; Figure 4). Lokhande et al. (2020) suggest that AMF has a strong binding affinity (-27.0441 kcal/mol) towards SARS-CoV-2-Mpro by the molecular docking analysis. Further, they reveal that AMF is highly stable and is of less conformational fluctuations with the Mpro enzyme through molecular dynamic simulations (Lokhande et al., 2020). Similarly, Ghosh et al. (2020) confirm that AMF interacts with two important catalytic residues (His41 and Cys145) of SARS CoV-2-Mpro, and exhibits higher binding affinity (-9.2 kcal/mol) towards Mpro than those of two well-known Mpro inhibitors N3 (-7.0 kcal/mol) and lopinavir (-7.3 kcal/mol). Molecular dynamics studies further reveals that AMF is of highly stability, less conformational fluctuations and shares a similar degree of compactness (Ghosh et al., 2020). Saravanan et al. (2020) find that AMF shows highly binding energy of -10.0 kcal/mol and stable interaction after binding with the SARS-COV2 main protease. AMF records −9.7 kcal/mol of binding energy against Mpro and interacts with target AAR by forming H bonds with Glu166 and other residues in the vicinity of catalytic site (Puttaswamy et al., 2020). AMF has a docking score of -7.766 kcal/mol which points out a strong bind with SARS-CoV-2 main protease (Mpro). AMF forms hydrogen bond (HB) interactions with Glu166, Thr25, His41 and Ser46 residues, and also forms a π-π stacking interaction with His41 residue (Patil et al., 2021). AMF exhibits a binding affinity of -8.1 kcal/mol and key amino acids including Asn151 and His246 are involved in the hydrogen bond (HB) interactions (Rameshkumar et al., 2021). In addition, AMF is also found to have strongly binding affinity (–9.4 kcal/mol) with SARS CoV-2 3CLpro, and can stabilize the three-dimensional conformations of 3CLpro after binding (Swargiary et al., 2020). There are also four docking studies targeting spike glycoprotein RBD of SARS-CoV-2. These studies reveal that AMF can strong bind with spike glycoprotein RBD of SARS-CoV-2 with the binding energies: -7.6 kcal/mol (Wei et al., 2020), -8.7 kcal/mol (Puttaswamy et al., 2020), -8.5 kcal/mol (Miroshnychenko and Shestopalova (2021)) and -10.2 kcal/mol (Rameshkumar et al., 2021). However, the binding sites of AMF are different in these studies. Wei et al. (2020) and Rameshkumar et al. (2021) suggest that AMF binds with the outside of the ACE2-binding region, while Miroshnychenko and Shestopalova, (2021). and Puttaswamy et al. (2020) reveal that AMF binds with the ACE2-binding region. Besides AMF binds with the main protease (-8.1 kcal/mol) and spike protein (-10.2 kcal/mol) of SARS-CoV-2, AMF can also bind with RNA-dependent RNA polymerase (RdRp) with a binding affinity of -8.1 kcal/mol (Rameshkumar et al., 2021). Altogether, the above-mentioned studies *in silico* approaches suggest that AMF could be a potential inhibitor of SARS-CoV-2 proteins (i.e., Mpro/3CLpro, RBD of Spike protein, and RNA-dependent RNA polymerase) and an effective drug candidate for SARS-CoV-2.

## 7 Bioavilability and Drug Delivery of Amentoflavone

AMF is a hydrophobic molecule and practically insoluble in water. To defeat the water insolubility and low bioavailability of AMF, some potential efficient drug delivery carriers which can wrap AMF inside are developed, such as the N-vinyl pyrrolidone-maleate-guerbet alcohol monoester polymer [P(NVP-MGAM)] micelles (Zhang et al., 2019), the amorphous solid dispersion (ASD) with polyvinylpyrrolidone K-30 (PVP K-30) (Chen et al., 2020a) and AMF-loaded vitamin E polyethylene glycol succinate (TPGS)/soluplus mixed micelles (Feng et al., 2020) (Table 5). These drug delivery carriers have effectively improved the solubility and bioavailability of AMF.

**TABLE 5:** various drug delivery carriers containing amentoflavone.

Carrier	Model system	Inference	References
N-vinyl pyrrolidone-maleate-guerbet alcohol monoester polymer [P(NVP-MGAM)]	KKAy insulin resistant diabetes mice models	P(NVP-MGAM)/AMF micelles enhance the oral bioavailability of amentoflavone, and is a potent drug for diabetes treatment	Zhang et al. (2019)
amorphous solid dispersion (ASD) with polyvinylpyrrolidone K-30	A549 xenograft-bearing mice models	ASD is an efficient drug delivery system, and reduce in tumor size and microvascular density occurred	Chen et al. (2020a)
TPGS/soluplus mixed micelles	A549 cells *in vitro*, Sprague–Dawley (SD) male rats *in vivo*	AMF-loaded mixed micelles have lower IC50 value to A549 cells in the cytotoxicity test, and increase metabolites in plasma and urine in rats	Feng et al. (2020)

P (NVP-MGAM)/AMF micelle is produced to load AMF into the P (NVPMGAM) micelle by the dialysis method (Zhang et al., 2019). Compared with AMF suspension group, P (NVP-MGAM)/AMF micelle group not only improves pharmacokinetic parameters, such as delaying the Tmax, prolonging the retention time in blood and increasing the area under the curve (AUC), but also increases tissue distribution. This result indicates that the P (NVP-MGAM)/AMF micelle is an efficient AMF delivery carrier which can slow AMF metabolism and enhance AMF bioavailability. Additionally, The accumulation of P (NVP-MGAM)/AMF micelle shows a better antidiabetic efficacy by activating the PPAR-γ and PI3K/Akt signaling pathway comparing with AMF suspension in KKAy insulin resistant diabetes mice (Zhang et al., 2019). As a windfall benefit, P (NVP-MGAM)/AMF micelle may be a potent drug for diabetes mellitus treatment.

Selaginella doederleinii (TBESD, containing five active ingredients: AMF, robustaflavone, 2″,3″-dihydro-3′, 3‴-biapigenin, 3′,3‴-binaringenin and delicaflavone) amorphous solid dispersion (TBESD-ASD) with polyvinylpyrrolidone K-30 (PVP K30) is successfully established by the solvent evaporation method. TBESD-ASD with PVP K-30 shows a higher dissolution rate and stability than free TBESD. Moreover, the absorption and bioavailability of TBESD-ASD are substantially higher than free TBESD by comparing the pharmacokinetic parameters (such as mean Cmax, MRT values, and AUC). In xenograft mice transplanted with A549 cells, the TBESD-ASD exhibits greater antitumor effect than free TBESD by blocking tumor angiogenesis (Chen et al., 2020a). These results demonstrate that ASD is an efficient drug delivery carrier for TBESD and can improve the bioavailability of TBESD.

AMF-loaded TPGS/soluplus mixed micelle is prepared by membrane hydration method (Zhao et al., 2017a; Feng et al., 2020). *In vitro*, AMF-loaded TPGS/soluplus mixed micelle shows higher toxicity to A549 cells than AMF. In rats, 14 metabolites including 11 in feces, 6 in urine, and 3 in plasma in AMF-loaded mixed micelle group are found, while only 3 metabolites in urine and no metabolites in plasma and bile of AMF group were found (Feng et al., 2020). TPGS/soluplus drug nanomicelle carrier successfully improves the bioavailability of AMF.

## 8 Clinical Prospective

In this review, we suggest that AMF, a natural biflavonoid compound with extensive pharmacological effects, is a potential drug candidate. Various studies have shown the potential application of AMF against dengue, herpes, candidiasis, chronic hepatitis and other infect diseases. In addition, AMF can inhibit the proteolytic/catalytic activity of SARS CoV-2 Mpro/spike protein/RdRp and might be a useful therapeutic drug to control SARS-CoV-2. AMF might be a potential therapeutic agent for prevention and/or treatment of UV and *γ*-irradiation induced damage. Furthermore, the neuroprotective effect of AMF is evident in its ability to against neurodegenerative diseases, including ischemic stroke, epilepsy, Parkinson’s disease, Alzheimer’s disease. AMF has also excellent potential therapeutic agent against bone diseases such as osteoporosis, rheumatoid arthritis, osteoarthritis. Numerous researches on AMF have revealed its cytotoxic potential against different cancers, such as HCC, breast cancer, osteosarcoma, bladder cancer, ovarian cancer, etc. AMF suppresses tumor pathological progress and metastasis *in vitro* and *in vivo* through several molecular mechanisms, including cell cycle arrest, apoptosis and autophagy induction, etc. AMF acts anti-cancer effect also by initiating p53 and inhibiting NF-κB, PI3K-AKT, ERK, and MAPK/mTOR signal pathways. Being a natural antioxidant and antibacterial agent in the food industry, AMF could be a potential use to improve the nutritional quality of food or processed food products.

Various animal researches strongly advocate the potential role of AMF in controlling tumor development, metabolic disorders, skeletal diseases and nerve protection. However, there is no clinical research investigation on the efficacy of AMF by now. Since AMF widely exists in nature, its utilization can greatly economize expenses related to growing diseases. As the improvement of delivery system, the absorption and bioavailability of AMF are significantly increased. In future, the preclinical and clinical studies are crucial for us to exploit the therapeutic potential of AMF and will help us to apply the active compound to the clinic.

## 9 Conclusion

This review discussed the multiple biological activities of AMF revealed in the past 40 years. AMF improves inflammation by inhibiting the activation of NF-KB signaling pathway and the downstream target genes. AMF protects neurological and skeletal diseases because of its anti-oxidative and anti-inflammatory activities. In addition, AMF restores the imbalance of lipid and carbohydrate metabolism and reverses DNA damage caused by radiation. AMF increases the expression of apoptosis and autophagy-related proteins, inhibits the expression of cell cycle, metastasis-associated proteins, and led to control cancer development. *In Silico*, AMF is forecasted to bind tightly with the spike, Mpro and RdRp proteins of SARS-CoV-2. This implies that AMF is a potential drug for the treatment of COVID-19.

In summary, AMF may be a broad and effective multifunctional active agent in disease therapy.

## Abbrevations

ACE2: angiotensin-converting enzyme 2
AMF: Amentoflavone
AMPK: AMP-activated protein kinase
Ask: aspartate kinase
ASK1: Apoptosis signal-regulating kinase 1
Bax: B-cell lymphoma 2 associated X
Bcl-2: B-cell lymphoma protein-2
BCR: B cell receptor
Bid: BH3 interacting-domain death agonist
BMMs: bone marrow-derived macrophages
CAT: Catalase
CDK1: cyclin-dependent kinase 1
C-FLIP: cellular Fasassociated protein with death domain-like interleukin 1 betaconverting enzyme inhibitory protein
COX-2: cyclooxygenase-2
CPA: Alpha-toxin
CRP: C reactive protein
Cyt-c: cytochrome c
EMT: epithelial-to-mesenchymal transition
ERK1/2: extracellular regulated protein kinases 1/2
ESR: Erythrocyte sedimentation rate
G-6-Pase: Glucose-6-phosphatase
GCK: Glucokinase
GPx: glutathione peroxidase
GSK-3: Glycogen synthase kinase-3
HB: Haemoglobin
HO-1: Hemeoxygenase 1
HMGB1: high mobility group box 1
iNOS: inducible nitric oxide synthase
IL-1β: Interleukin 1β
IL-6: Interleukin-6
IL-8: Interleukin-8
IC50: inhibitory concentration 50%
JNK1/2: Jun N-terminal protein kinase 1 and 2
mTOR: mammalian target of rapamycin
MAPK: mitogen-activated protein kinase
MCL-1: myeloid cell leukemia sequence 1
MDA: malondialdehyde
MMP-2: matrix metalloproteinase 2
MMP-9: matrix metalloproteinase 2
MMP: mitochondrial membrane potential
MPTP: methyl-4-phenyl-1,2,3,6-tetrahydropyridine
NF-κB: nuclear factor κB
NO: nitric oxide
NLRP3: nucleotide oligomerization domain-like receptor protein 3
Nrf2: Nuclear factor erythroid 2-related factor 2
PARP: poly (adenosine diphosphate-ribose) polymerase
PEPCK: Phosphoenolpyruvate carboxylase kinase; PFK-1:6-Phosphofructokinase
PFO: perfringolysin O
PGE2: Prostaglandin E2
PI3K: Phosphatidyl inositol 3-kinase
PK: Pyruvate kinase
PMNs: polymorphonuclear neutrophils
PPARγ: peroxisome proliferator-activated receptor γ
ROS: reactive oxygen species
RNS: reactive nitrogen species
SOCS3: suppressor of cytokine signaling 3
SOD: superoxide dismutase
TNF-α: tumor necrosis factor α
TNFAIP2: tumor necrosis factor alpha-induced protein 2
uPA: urokinasetype plasminogen actvator
UGM: uridine diphosphogalactofuranose (UDP)-galactopyranose mutase
VEGF: vascular endothelial growth factor
